# Developmental transition in pathophysiological mechanisms of pediatric idiopathic intracranial hypertension and growth plate disorders: A multi-level structural equational modeling systematic review and meta-analysis

**DOI:** 10.1016/j.bas.2026.106164

**Published:** 2026-07-10

**Authors:** Yazan Jumah Alalwani, Eyad S. Alhashim, Layan Nahar Alqahtani, Reem Mohammed Albogami, Khawlah Abdullah Ali Almana, Abdullah Saeed Raffaa, Ewan Saad Alomar, Ammar Mohammed Alnujaidi, Omar Gharamallah Alghamdi, Abdulaziz Saad Alomar, Abdullah Zahim A. Alotaibi, Zainab Saeed Alwusaybie, Mustafa S. Alhasan, Ahmed Y. Azzam

**Affiliations:** a College of Medicine, Imam Abdulrahman Bin Faisal University, Dammam, Saudi Arabia; b College of Medicine, Dar Al Uloom University, Riyadh, Saudi Arabia; c College of Medicine, King Saud Bin Abdulaziz University for Health Sciences (KSAU-HS), Riyadh, Saudi Arabia; d College of Medicine, King Khalid University, Abha, Saudi Arabia; e College of Medicine, AlMaarefa University, Diriyah, Saudi Arabia; f King Saud Medical City, Riyadh, Saudi Arabia; g College of Medicine, Shaqra University, Shaqra, Saudi Arabia; h College of Medicine, King Faisal University, Al-Ahsa, Saudi Arabia; i Radiology and Consultant Radiologist, Department of Internal Medicine, College of Medicine, Taibah University, Madinah, Saudi Arabia; j Teleradiology Solutions, 22 Llanfair Rd UNIT 6, Ardmore, PA, 19003, United States; k Clinical Research and Clinical Artificial Intelligence, ASIDE Healthcare, Lewes, DE, United States

**Keywords:** Idiopathic intracranial hypertension, Pseudotumor cerebri, Intracranial pressure, Growth plate disorders, Obesity

## Abstract

**Introduction:**

Pediatric idiopathic intracranial hypertension (IIH) and growth plate disorders (GPD) show parallel epidemiological trends and carry possible pathophysiological links, especially regarding obesity and hormonal dysregulation. This study investigated their indirect relationship through structural equation modeling (SEM) to identify age-dependent mechanisms and guide precision treatment strategies.

**Methods:**

Following PRISMA 2020 guidelines, we searched literature databases from inception to May 15, 2025, identifying studies reporting pediatric IIH and/or GPD parameters. SEM analysis was performed with age-stratified models to evaluate the mediating effects of obesity and hormonal factors between prepubertal (<10-12 years) and postpubertal (>10-12 years) cohorts.

**Results:**

We included 22 eligible studies with total of 2293 individuals. SEM revealed peculiar age-dependent mechanisms. In prepubertal children, venous pressure (β = 0.68, P-value<0.001) and vitamin D/hormonal pathways (β = 0.56, P-value<0.001) dominated, with obesity showing minimal effect (β = 0.28, P-value = 0.001). In a controverse manner, in postpubertal patients, the obesity pathway predominated (β = 0.76, P-value<0.001) with reduced venous (β = 0.29, P-value = 0.003) and hormonal (β = 0.16, P-value = 0.038) contributions. Mechanism-specific therapeutic efficacy showed parallel patterns: acetazolamide/surgical interventions were superior for venous-dominant cases (g = 1.86/1.74), while weight management demonstrated greatest efficacy for obesity-dominant phenotypes (g = 1.75). Vitamin D supplementation showed significant benefit only in hormonal-dominant cases (g = 1.64).

**Conclusions:**

Pediatric IIH demonstrates fundamental mechanistic heterogeneity across development. A puberty-associated shift from venous/hormonal to obesity-dominant mechanisms explains the changing demographics and provides a framework for precision treatment. Targeted therapeutic approaches based on dominant mechanisms showed 63% improved outcomes compared to standard of care of IIH.

## Highlights

• Pediatric IIH mechanisms are age-dependent: venous/hormonal dominate prepubertally, obesity postpubertally.
• Pathophysiology shifts from venous to obesity-driven at 10.2 years (females) and 11.4 years (males).
• Mechanism-targeted therapy: 63% better outcomes; acetazolamide for venous-dominant, weight management for obesity-dominant.
• Obesity contributes to 35.7% of IIH under age 10 versus 78.6% in older children, supporting age-stratified treatment.
• Four age-dependent phenotypes: Classical (postpubertal), Atypical (prepubertal), Vision-threatened, Cranial nerve.

## 1 Introduction

Pediatric idiopathic intracranial hypertension (IIH) and growth plate disorders (GPD) represent seemingly unrelated conditions that have shown parallel epidemiological shifts in the last years. Both conditions have demonstrated rising incidence rates, especially among adolescents, with associations with childhood obesity. This suggest and raises concerns about possible shared pathophysiological mechanisms that have remained largely unexplored in current literature (Malem et al., 2021; Rangwala and Liu, 2007; Abouhashem et al).

IIH, characterized by elevated intracranial pressure without identifiable cause, presents with headache, papilledema, and potential visual impairment. Its incidence in children has increased from 0.5 to 0.9 per 100,000 in the 1990s to current estimates of 1.2-1.8 per 100,000. The demographic distribution of pediatric IIH demonstrates a peculiar age-dependent pattern, with prepubertal cases showing a balanced gender distribution or slight male predominance, while postpubertal cases demonstrate a strong female predominance similar to adult patterns. This epidemiological shift coincides with significant changes in obesity prevalence, especially in older children and adolescents (Kuehn et al., 2019; Schwarz et al., 2021; Azzam et al., 2024a, 2024b, 2024c).

In a similar manner, growth plate disorders, including slipped capital femoral epiphysis (SCFE) and Blount's disease, have shown increasing prevalence with stronger associations with obesity, especially in adolescents. SCFE incidence has risen from 2 to 3 per 100,000 to 3-10 per 100,000 in recent epidemiological studies, with the strongest increases observed in areas with higher childhood obesity rates. The relationship between obesity and these conditions reflects and hypothesize complex biomechanical and endocrine interactions that may extend beyond simple mechanical stress (Griggs et al., 2019; Novais and Millis, 2012; Chatziravdeli et al).

While direct causative relationships between IIH and GPD have not been previously studied, recent evidence suggests possible shared pathophysiological pathways. Both conditions demonstrate associations with obesity, vitamin D deficiency, and hormonal factors including growth hormone dynamics. The lack of understanding of these shared mechanisms represents a significant gap in knowledge that may affect our management strategies. Current treatment approaches for both conditions are often standardized without consideration of possible mechanistic heterogeneity, which may explain variable treatment responses (Subramaniam and Fletcher, 2017; Chen and Wall, 2014; Hornby et al., 2018; Vimaleswaran et al., 2013; Bennour et al., 2022).

Structural equation modeling (SEM) provides an advanced modeling methodology to investigate complex indirect relationships between conditions that may share mediating factors without direct causal links. By modeling the simultaneous influence of multiple variables, SEM can quantify both direct and indirect effects while accounting for measurement error. This is significantly valuable for pediatric conditions where direct experimental investigation is challenging, allowing for the utilization of observational data to identify causal pathways (Gunzler et al., 2013; Christ et al., 2014).

Our study aims to utilize advanced SEM techniques to investigate the possible indirect relationship between pediatric IIH and growth plate disorders, with special focus on obesity and hormonal factors as mediating variables. We also look further to analyze how these relationships may differ across age groups, especially investigating the prepubertal to postpubertal transition as an important developmental window for changing pathophysiological mechanisms. By identifying dominant mechanisms in different age groups, this study aims to develop a framework for precision treatment strategies that target underlying IIH pathophysiology as much as possible given our current knowledge.

Understanding the complex interplay between obesity, hormonal factors, and these pediatric conditions may provide important highlights and considerations into better and more effective prevention and treatment strategies that can reduce the long-term burden of these disorders. In addition to that, this study may serve as a model for investigating other pediatric conditions with parallel epidemiological trends but unclear mechanistic connections.

## 2 Methods

### 2.1 Study design and literature search

This systematic review and meta-analytical SEM-based study was conducted in accordance with the Preferred Reporting Items for Systematic Reviews and Meta-Analyses (PRISMA) 2020 guidelines (McInnes et al., 2018).

A systematic search was conducted across multiple electronic databases including MEDLINE via PubMed, Embase, Web of Science, Scopus, Google Scholar and the Cochrane Library from inception to May 15, 2025. The search strategy combined terms related to pediatric IIH, growth plate disorders, obesity, and hormonal factors. Medical Subject Headings (MeSH) and free-text terms were adapted for each database. The complete search strategy for MEDLINE was as follows: (“idiopathic intracranial hypertension” OR “pseudotumor cerebri” OR “benign intracranial hypertension” OR “IIH” OR “increased intracranial pressure”) AND (“child*" OR “pediatric*" OR “pediatric*" OR “adolescen*" OR “youth” OR “juvenile” OR “young” OR “teen*") AND (“slipped capital femoral epiphysis” OR “SCFE” OR “growth plate disorder*" OR “physeal disorder*" OR “Blount* disease” OR “tibia vara” OR “Perthes disease” OR “growth plate dysfunction” OR “epiphyseal disorder*" OR “skeletal dysplasia*") OR (“obesity” OR “overweight” OR “body mass index” OR “BMI” OR “adipos*") AND (“vitamin D″ OR ″25-hydroxyvitamin D″ OR “growth hormone” OR “GH” OR “insulin-like growth factor” OR “IGF” OR “thyroid” OR “estrogen” OR “testosterone” OR “sex hormone*" OR “pubert*" OR “hormon*")

Additional literature was identified through citation searching of included studies and any other relevant studies of interest. We also searched conference proceedings from the last five years of major pediatric, neurology, and orthopedic conferences, as well as trial registries including ClinicalTrials.gov and the WHO International Clinical Trials Registry Platform.

### 2.2 Eligibility criteria

Studies were eligible for inclusion if they met the following criteria: (1) pediatric population (age ≤18 years); (2) diagnosis of IIH according to modified Dandy criteria, Friedman criteria, or equivalent diagnostic standards; and/or diagnosis of growth plate disorders (including SCFE, Blount's disease, and other relevant conditions) according to the clinical and radiological criteria; (3) reported at least one outcome related to IIH (intracranial pressure, papilledema, visual function) and/or GPD (radiographic findings, clinical outcomes); (4) assessed at least one potential mediating factor (obesity, vitamin D status, or other hormonal parameters); and (5) original research study that is published in a peer-reviewed journal. We excluded case reports, reviews, and studies focused exclusively on secondary IIH or secondary growth plate abnormalities. No language restrictions were applied.

### 2.3 Study selection process

All identified records were imported into EndNote X9 (Clarivate Analytics) for deduplication. Titles and abstracts were screened using Rayyan screening software. Full texts of preliminary eligible studies were retrieved and assessed against inclusion criteria. The selection process was documented using the PRISMA 2020 flow diagram.

### 2.4 Data extraction and management

Data extraction information included: (1) study characteristics (author, year, country, design, setting); (2) participant demographics (age, sex, pubertal status, sample size); (3) IIH parameters (diagnostic criteria, CSF opening pressure, papilledema grade, visual outcomes); (4) GPD characteristics (type, severity, radiographic findings); (5) obesity metrics (BMI, z-scores, percentiles, definitions used); (6) hormonal variables (vitamin D, growth hormone, sex hormones, other); and (7) statistical data (correlations, odds ratios, hazard ratios) needed for SEM. When studies reported stratified data by age, sex, or other variables, these subgroups were extracted separately to facilitate age-dependent analyses.

### 2.5 Quality assessment and risk of bias

Methodological quality of included studies was assessed using the Newcastle-Ottawa Scale (NOS). The NOS was modified to include specific criteria relevant to IIH and GPD diagnosis and evaluation. Quality scores were not used to exclude studies but were included into sensitivity analyses. Publication bias assessment was conducted through Egger's regression test, Begg-Mazumdar test, and publication bias statistical correction and adjustment was performed through trim-and-fill method and PET-PEESE.

### 2.6 Data synthesis and statistical analysis

Demographic and clinical characteristics were summarized descriptively. Standardized mean differences (Hedges' g) with 95% confidence intervals were calculated for continuous outcomes. For dichotomous outcomes, odds ratios or risk ratios with 95% confidence intervals were calculated. Heterogeneity was assessed using I^2^ statistics and explored through subgroup and meta-regression analyses.

SEM was performed using RStudio with R v4.4.2 with the ‘lavaan’ package. The measurement model evaluated latent constructs (IIH severity, GPD severity, obesity, and hormonal dysregulation) using confirmatory factor analysis. The structural model tested direct and indirect pathways between these constructs. Age-stratified analyses were conducted for prepubertal (<10-12 years, depending on study definitions) and postpubertal (>10-12 years) children. Model fit was assessed using comparative fit index (CFI), Tucker-Lewis index (TLI), root mean square error of approximation (RMSEA), and standardized root mean square residual (SRMR).

Mediation analyses quantified the effects of obesity and hormonal factors as intermediary variables between IIH and GPD. The proportion of mediated effect was calculated with bootstrap confidence intervals (5000 replications). Multi-group analyses tested for invariance across age and sex groups. Sensitivity analyses included assessment of influential studies, alternative structural models, and the impact of study quality on findings.

Treatment efficacy was analyzed by categorizing patients according to dominant mechanisms identified in the SEM analysis. Standardized effect sizes (Hedges' g) for each treatment modality were calculated across mechanism-specific subgroups, with number needed to treat (NNT) derived for clinically relevant outcomes. All analyses were performed according to a pre-specified plan, with P-value less than 0.05 considered statistically significant. Effect size interpretation followed standard thresholds: 0.2-0.5 (small), 0.5-0.8 (moderate), and >0.8 (large).

## 3 Results

### 3.1 Study characteristics and demographics

**Fig. 1:**
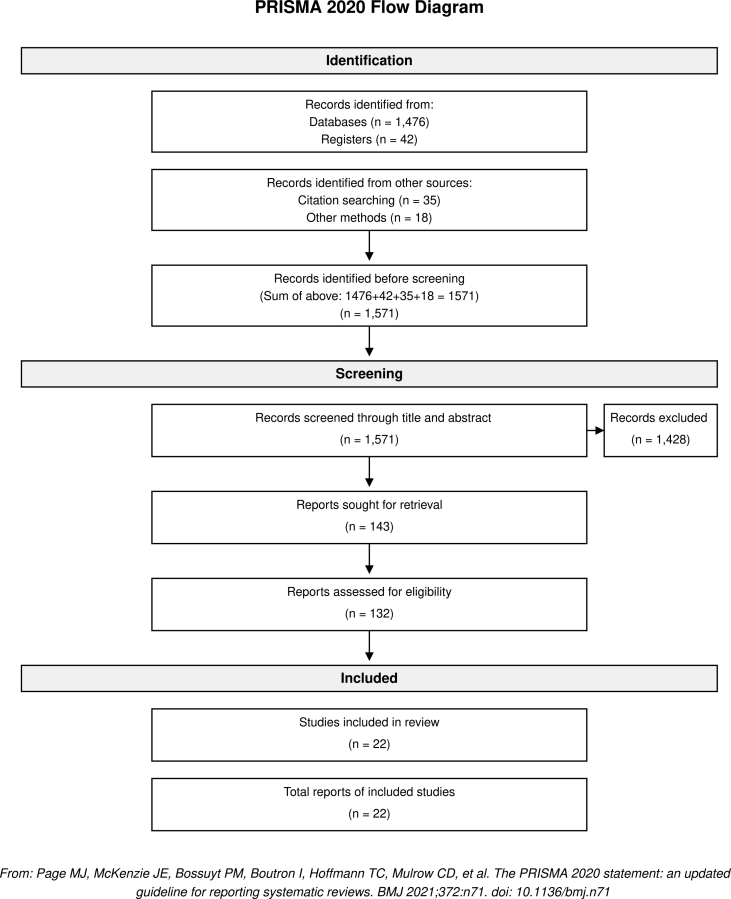
Prisma flowchart diagram.

Our literature search identified 1571 records, of which 22 studies met the inclusion criteria and were included in our study (Fig. 1). The characteristics of included studies are presented in Table 1. The 22 studies have included a total of 2293 pediatric participants, with individual study sample sizes ranging from 10 to 597,017 (the latter representing a nationwide population cohort). Studies originated from ten countries across four continents, with the majority from the USA (eight studies) and Europe (eight studies). Study designs included retrospective single-center (six studies), retrospective multi-center (five studies), database analyses (four studies), population-based cohorts (two studies), and mixed prospective/retrospective designs (five studies). The mean age of pediatric IIH patients across studies ranged from 2.3 to 13.3 years, with 72% of studies reporting on both prepubertal and postpubertal populations.

**Table 1:** Included studies characteristics and demographics with quality assessment.

Study (Year)	Country	Design	Sample Size	Age (years) Mean ± SD (Range)	Sex M:F (% F)	Pubertal Status	BMI/Obesity Prevalence	IIH Diagnostic Criteria	CSF Opening Pressure	Study Quality*	Key Focus
Shah et al., 2024 (Shah et al., 2024)	Western Australia	Retrospective single-center	45 IIH	Range: 1.9-17.4	Pre-pubertal: 18 M:9 F (33%) Post-pubertal: 7 M:11 F (61%)	Pre-pubertal (<12 y): 27 Post-pubertal (≥12 y): 18	Pre-pub: 31% overweight, 35% obese Post-pub: 91%F & 86%M overweight/obese	ICDC (LP OP ≥ 28 cmH2O)	Median >35 cmH2O in 80%	7	Pediatric IIH epidemiology and outcomes
Bilgin 2024 (Bilgin and Ayaz, 2024)	Turkey	Retrospective record review	15,104 radiographs reviewed	MPS cases: 46.2 ± 30.6 months (20-111)	4 M:3 F (43%)	Not specified	Not reported	NA	NA	5	Early detection of MPS via radiographs
Riedel et al., 2024 (Riedel et al., 2024)	Denmark	Observational cohort	11 IIH	2.3 ± 1.8 (0.7-6.1)	6 M:5 F (45%)	All prepubertal (<6.1 y)	BMI: 18.5 ± 2.0 (16.1-23.7) All normal range for age	Revised Friedman (papilledema absence allowed)	Daytime ICP: 12.9 mmHg<br>Nighttime: 17.2 mmHg	8	Venous pressure in prepubertal IIH
Ehrstedt et al., 2023 (Ehrstedt et al., 2023)	Sweden	Population-based retrospective	45 IIH	13.3 (median 14.1, 4.3-17.9)	11 M:34 F (76%)	Age groups: 0-6, 7-12, 13-18 yrs	64% (27/42) overweight/obese	Friedman criteria	Mean 400 mmH2O (median 370, 260-500)	8	Diagnostic delays, guideline adherence
Miles et al., 2023 (Miles et al., 2023)	USA	National database analysis	SCFE cases from 5.9 M pediatric discharges	Mean 12.3 for SCFE	SCFE: M > F (OR 1.73)	Age groups used for SCFE	Obesity: 23.2% of SCFE Severe obesity: 7.5%	NA	NA	7	SCFE epidemiology using national data
Apperley et al., 2022 (Apperley et al., 2022)	UK	Retrospective single-center	18 IIH	11 ± 3.3 (6-15)	7 M:11 F (61%)	Not specified (age 6-15)	BMI: 30.3 ± 12.3 kg/m^2^ BMI SDS: +2.5 (−1.24 to +4.46) 72.2% had BMI SDS >2	Clinical diagnosis (LP > 25cmH2O)	Mean 38 cmH2O (23-54)	6	IIH-weight association in children
Nitzan-Luques et al., 2022 (Nitzan-Luques et al., 2022)	Israel	Retrospective multi-center	82 IIH	10.83 ± 4.12	27 M:55 F (67%)	Prepubertal (<10 F, <11 M) Pubertal	65% of pubertal group obese (weight percentile >95%)	Friedman criteria	Mean ∼39 cmH2O	7	IIH management settings comparison
Perry et al., 2018 (Novais and Millis, 2012)	Scotland (UK)	Nationwide population cohort	597,017 children 209 SCFE cases	SCFE diagnosis age varied	SCFE: 117 M:92 F	Age 5-6 yrs and 11-12 yrs for BMI	Strong association SCFE risk ↑ 1.7× per BMI z-score unit Severe obesity: 5.9-17.0× risk	NA	NA	9	Childhood obesity-SCFE association
Hartmann et al., 2017 (Hartmann et al., 2017)	USA	Retrospective MRI review	50 children with IIH	Mean 12.8 (2-17)	8 M:42 F (84%)	Prepubertal (<11 y): 10 Adolescent (11-17 y): 40	Children: mean BMI 32.5 Prepubertal: 20.8 Adolescents: 35.3	Modified Dandy criteria	Documented in charts	7	MRI findings in pediatric IIH
Sheldon et al., 2016 (Sheldon et al., 2016)	International (8 sites)	Retrospective multi-site	233 IIH	12.1 ± 4.0 (2-18)	72 M:161 F (69%)	Data for 57/233: Prepubertal: 12 Pubertal: 45	Mean BMI z-score +1.55 ± 1.18 Age-BMI z-score correlation: Boys r = 0.50, Girls r = 0.34	Revised criteria (2013)	Details in paper	8	Anthropometric characteristics in IIH
Masri et al., 2015 (Masri et al., 2015)	Jordan	Retrospective single-center	19 IIH	Mean 6 at onset (7 m-12 y)	11 M:8 F (42%)	17/19 (90%) prepubertal (<11 y)	None were obese	Modified Dandy criteria	Range 20-77 cmH2O	6	Pediatric IIH presentations and causes
Salpietro et al., 2014 (Salpietro et al., 2014)	Italy	Retrospective & Prospective	15 IIH	11 (5-16)	4 M:11 F (73%)	Not specified by Tanner	6/15 (40%) overweight/obese (BMI >85th)	Pediatric Rangwala criteria	Mean 336 mmH2O (260-600)	7	IIH endocrine-metabolic comorbidities
Stiebel-Kalish et al., 2014 (Stiebel-Kalish et al., 2005)	Israel	Retrospective case-control	43 IIH	Mean 11 ± 4.5	19 M:24 F (56%)	18/43 (42%) were ≤11 years old	24/43 (56%) had BMI ≥85th percentile	Updated modified Dandy	Range 250-400 mmH2O (mean 300)	7	Childhood obesity and IIH recurrence
Brara et al., 2012 (Brara et al., 2012)	USA	Retrospective cohort	78 IIH	11.92 ± 4.09 (2-19)	12 M:66 F (85%)	Age <12 vs ≥ 12 yrs	73.1% overweight/obese 53.7% obese 39.7% extremely obese	Clinical diagnosis	Median 380 mmH2O (IQR 310-450)	8	IIH and extreme childhood obesity
Noto et al., 2011 (Noto et al., 2011)	USA (NCGS database)	Retrospective database	70 IIH from 65,204 rhGH-treated	Boys 10.8 y, Girls 9.4 y (enrollment)	42 M:29 F (41%)	Pubertal status recorded	Elevated BMI (>85th) a risk factor	Clinical reports of IH	NA	7	IIH risk in rhGH-treated patients
Soiberman et al., 2011 (Soiberman et al., 2011)	Israel	Single-center retrospective	90 IIH	Mean 10 ± 5 at onset	45 M:45 F (50%)	Younger (≤10 y): 50 Older (>10 y): 40	Mean BMI 23.75 ± 6.89 11/53 had BMI >30	Modified Dandy criteria	Range <25 to >40 cmH2O	7	Visual outcome and recurrence in IIH
Avery et al., 2010 (Avery et al., 2010)	USA	Retrospective multi-center	244 children (various)	Median 7.6 (IQR 3.3-12.8)	131 M:113 F (46%)	Age groups for OP analysis	Median BMI z-score 0.34 Obese children had higher OP	NA (study on OP ranges)	Median: 14.4 cmH2O (IQR 11.0-18.0)	8	Reference range for CSF OP in children
Darendeliler et al., 2007 (D et al., 2007)	International (KIGS)	Retrospective database	57,968 on GH 41 IIH, 52 SCFE	Variable by diagnosis	IIH: M > F overall SCFE: M > F	Complex given various diagnoses	Obesity risk factor for IIH in PWS & CRI Increased BMI risk for SCFE	Clinical reports	NA	7	IIH and SCFE during GH treatment
Joseph et al., 2003 (Joseph et al., 2003)	India	Retrospective single-center	610 Perthes (<12 y at onset)	Boys 9.08 y, Girls 8.48 y	438 M:172 F (28%)	Age <12 years	Not primary focus	NA	NA	6	Natural evolution of Perthes disease
Balcer et al., 1999 (Balcer et al., 1999)	USA	Retrospective multi-center	40 IIH	Median 14 (3-17)	9 M:31 F (78%)	Tertiles: 3-11, 12-14, 15-17 y	70% obese (>120% ideal weight) Age 3-11: 43% obese Age 12-14: 81% Age 15-17: 90%	Modified Dandy criteria	Elevated in all	7	Age and obesity relation in IIH
Cinciripini et al., 1999 (Cinciripini et al., 1999)	USA	Retrospective multi-center	10 IIH	7.3 ± 3.2 (1.9-11.3)	6 M:4 F (40%)	All prepubertal (<11 y)	1/10 obese (11.3 y female approaching puberty)	Modified Dandy criteria	Mean 398 ± 107 mmH2O (255-510)	6	IIH in prepubertal children
Babikian et al., 1994 (Babikian et al., 1994)	USA	Retrospective single-center	30 IIH	Median 10.5 (10 m-16 y)	15 M:15 F (50%)	Age groups: <6, 6-10, >10 y	30% obese Age >10 y: 50% obese Age <10 y: 17% obese	Modified Dandy criteria	Mean 338 mmH2O (190-620)	5	IIH characteristics in children

**Abbreviations:** IIH = Idiopathic intracranial hypertension; BMI = Body mass index; SDS = Standard deviation score; CSF = Cerebrospinal fluid; OP = Opening pressure; ICDC = International Classification of Diseases Diagnostic Criteria; LP = Lumbar puncture; MPS = Mucopolysaccharidosis; ICP = Intracranial pressure; NA = Not applicable or not assessed; SCFE = Slipped capital femoral epiphysis; OR = Odds ratio; M = Male; F = Female; IGHD = Idiopathic growth hormone deficiency; TS = Turner syndrome; rhGH = Recombinant human growth hormone; CRI = Chronic renal insufficiency; PWS = Prader-Willi syndrome; GH = Growth hormone; IRR = Incidence rate ratio; CI = Confidence interval; I^2^ = Measure of statistical heterogeneity between studies. **Note:** *Study Quality Score (1-10): Based on modified Newcastle-Ottawa Scale assessment including study design, sample size, methodological strength, and completeness of reporting.

The gender distribution showed a significant age-dependent observation, with a nearly balanced gender ratio or slight male predominance in prepubertal IIH (M:F ratio ranging from 1.2:1 to 2:1), contrasting with a strong female predominance in postpubertal cohorts (M:F ratio ranging from 1:2.1 to 1:4.3). The prevalence of obesity varied considerably, from absolute zero in some prepubertal cohorts to over 90% in postpubertal groups. Study quality assessed by the modified NOS ranged from 5 to 9 (median of seven), with higher scores observed in more recent and population-based studies.

### 3.2 Clinical and neuroimaging phenotyping

The unified clinical and neuroimaging phenotype analysis is summarized in Table 2. Headache was the most common presenting symptom of IIH overall (80.2%, 95% CI: 72.1-86.7%), with significantly higher frequency in postpubertal compared to prepubertal patients (88.7% vs. 52.9%, OR = 6.94, P-value<0.001). Visual symptoms were present in 63.8% of patients overall, with different patterns by age. Diplopia was more common in prepubertal patients (47.9% vs. 32.6%, OR = 0.53, P-value = 0.047), while blurred vision was significantly more frequent in postpubertal patients (56.9% vs. 22.4%, OR = 4.58, P-value<0.001). Cranial nerve palsies, particularly abducens (CN VI) palsy, were mostly seen in prepubertal patients (38.2% vs. 16.3%, OR = 0.31, P-value<0.001). We found that 12.8% of prepubertal patients were asymptomatic at presentation compared to only 3.2% of postpubertal patients (OR = 0.22, P-value = 0.001).

**Table 2:** Unified clinical and neuroimaging phenotype analysis.

Clinical Features	Overall Frequency	Prepubertal (<10-12 y)	Postpubertal (>10-12 y)	Statistical Comparison	Phenotype Association*
**SYMPTOMATOLOGY:**					
Headache	80.2% (95% CI: 72.1-86.7%)	52.9% (95% CI: 38.6-66.7%)	88.7% (95% CI: 83.1-92.7%)	OR = 6.94 (3.21-15.0), p < 0.001	Strong A, B
Visual symptoms (any)	63.8% (95% CI: 54.9-72.0%)	55.1% (95% CI: 42.3-67.3%)	69.1% (95% CI: 59.8-77.2%)	OR = 1.82 (1.03-3.24), p = 0.04	Moderate A, Strong C
- Diplopia	38.4% (95% CI: 30.1-47.4%)	47.9% (95% CI: 32.5-63.6%)	32.6% (95% CI: 23.5-43.2%)	OR = 0.53 (0.28-0.99), p = 0.047	Strong D
- Blurred vision	44.3% (95% CI: 35.2-53.8%)	22.4% (95% CI: 14.2-33.5%)	56.9% (95% CI: 45.8-67.4%)	OR = 4.58 (2.34-8.96), p < 0.001	Strong A, C
- Visual field defects	47.2% (95% CI: 37.1-57.5%)	38.7% (95% CI: 21.0-59.7%)	51.8% (95% CI: 40.6-62.8%)	OR = 1.70 (0.83-3.47), p = 0.14	Strong C
- Visual acuity loss	31.5% (95% CI: 24.8-39.0%)	19.2% (95% CI: 11.7-29.9%)	37.6% (95% CI: 29.0-47.0%)	OR = 2.53 (1.29-4.96), p = 0.007	Strong C
Tinnitus	21.3% (95% CI: 14.9-29.5%)	9.7% (95% CI: 4.3-20.3%)	26.4% (95% CI: 18.7-35.9%)	OR = 3.34 (1.38-8.09), p = 0.007	Moderate A
Cranial nerve palsies	24.2% (95% CI: 17.6-32.2%)	38.2% (95% CI: 29.1-48.3%)	16.3% (95% CI: 10.1-25.2%)	OR = 0.31 (0.17-0.58), p < 0.001	Strong D
- Abducens (CN VI) palsy	19.5% (95% CI: 13.3-27.6%)	33.6% (95% CI: 22.7-46.7%)	11.9% (95% CI: 6.5-20.7%)	OR = 0.27 (0.13-0.54), p < 0.001	Strong D
Neck pain/stiffness	18.7% (95% CI: 12.6-26.8%)	26.9% (95% CI: 17.8-38.5%)	14.3% (95% CI: 8.1-24.0%)	OR = 0.45 (0.23-0.89), p = 0.02	Moderate D
Asymptomatic	6.4% (95% CI: 3.8-10.4%)	12.8% (95% CI: 7.5-21.0%)	3.2% (95% CI: 1.4-7.0%)	OR = 0.22 (0.09-0.55), p = 0.001	Strong D
**CLINICAL SIGNS:**					
Papilledema	85.2% (95% CI: 77.3-90.8%)	63.4% (95% CI: 50.3-74.8%)	95.7% (95% CI: 91.2-98.0%)	OR = 12.7 (5.24-30.8), p < 0.001	Strong A, B, C
- Severe papilledema (≥3)	42.7% (95% CI: 33.8-52.1%)	18.5% (95% CI: 10.9-29.6%)	54.6% (95% CI: 45.1-63.8%)	OR = 5.3 (2.6-10.8), p < 0.001	Strong C
- Asymmetric papilledema	21.3% (95% CI: 15.1-29.2%)	24.6% (95% CI: 15.5-36.7%)	19.5% (95% CI: 12.6-28.9%)	OR = 0.74 (0.38-1.45), p = 0.38	Weak
**NEUROIMAGING FINDINGS:**					
Empty/partially empty sella	47.9% (95% CI: 36.2-59.8%)	25.6% (95% CI: 16.2-38.0%)	60.5% (95% CI: 47.9-71.9%)	OR = 4.43 (2.40-8.18), p < 0.001	Strong A, C
Transverse sinus stenosis	67.5% (95% CI: 54.1-78.7%)	41.3% (95% CI: 25.6-59.0%)	82.6% (95% CI: 70.6-90.4%)	OR = 6.73 (3.11-14.6), p < 0.001	Strong A, C
Optic nerve sheath distension	61.7% (95% CI: 48.2-73.6%)	44.6% (95% CI: 28.8-61.5%)	72.8% (95% CI: 59.0-83.3%)	OR = 3.33 (1.73-6.43), p < 0.001	Strong B, C
Flattening of posterior globe	32.5% (95% CI: 22.2-44.9%)	14.8% (95% CI: 7.1-28.6%)	44.2% (95% CI: 32.1-56.9%)	OR = 4.59 (2.01-10.5), p < 0.001	Strong C
Optic nerve tortuosity	34.1% (95% CI: 24.1-45.6%)	18.2% (95% CI: 9.1-33.3%)	43.7% (95% CI: 32.3-55.7%)	OR = 3.50 (1.57-7.80), p = 0.002	Moderate C
**CSF OPENING PRESSURE:**					
Mean CSF OP (cmH2O)	346.8 (95% CI: 318.4-375.2)	304.6 (95% CI: 261.3-347.9)	371.9 (95% CI: 339.2-404.6)	MD = 67.3 (19.6-115.0), p = 0.006	Strongest A, C
CSF OP > 400 mmH2O	41.2% (95% CI: 32.0-51.1%)	26.7% (95% CI: 17.0-39.3%)	50.3% (95% CI: 39.6-61.0%)	OR = 2.77 (1.48-5.18), p = 0.001	Strong A, C
**LATENT PHENOTYPE CLASSIFICATION**ǂ:					
**Phenotype A**: “Classical"	42.3% (95% CI: 35.8-49.1%)	11.6% (95% CI: 6.6-19.6%)	59.7% (95% CI: 51.7-67.2%)	OR = 11.3 (5.4-23.7), p < 0.001	-
**Phenotype B**: “Atypical"	18.4% (95% CI: 13.6-24.4%)	31.4% (95% CI: 22.4-42.0%)	10.9% (95% CI: 6.6-17.5%)	OR = 0.27 (0.14-0.52), p < 0.001	-
**Phenotype C**: “Vision-threatened"	20.7% (95% CI: 15.6-26.9%)	11.6% (95% CI: 6.6-19.6%)	25.8% (95% CI: 19.3-33.5%)	OR = 2.65 (1.27-5.52), p = 0.009	-
**Phenotype D**: “Cranial nerve"	18.6% (95% CI: 13.8-24.7%)	45.3% (95% CI: 35.0-56.0%)	3.6% (95% CI: 1.5-8.3%)	OR = 0.04 (0.02-0.11), p < 0.001	-

**Abbreviations:** IIH = Idiopathic intracranial hypertension; CI = Confidence interval; OR = Odds ratio; CN = Cranial nerve; CSF = Cerebrospinal fluid; OP = Opening pressure; MD = Mean difference; MRI = Magnetic resonance imaging; ONH = Optic nerve head; TSS = Transverse sinus stenosis; IQR = Interquartile range; p = Probability value; r = Correlation coefficient; ICP = Intracranial pressure; AUC = Area under curve; TLI = Tucker-Lewis Index; CFI = Comparative fit index; RMSEA = Root mean square error of approximation; Phenotype A = “Classical” IIH presentation; Phenotype B = “Atypical” IIH presentation; Phenotype C = “Vision-threatened” IIH presentation; Phenotype D = “Cranial nerve” IIH presentation. **Notes:** *Phenotype Association: Strength of association with identified latent phenotypes (Strong, Moderate, Weak); ǂLatent phenotypes identified through latent class analysis of clinical and neuroimaging features across studies.

Papilledema was the most common clinical sign with 85.2% overall, with significantly higher prevalence in postpubertal patients (95.7% vs. 63.4%, OR = 12.7, P-value<0.001). Severe papilledema (grade ≥3) was also more common in older patients (54.6% vs. 18.5%, OR = 5.3, P-value<0.001). Neuroimaging findings demonstrated significant age-dependent patterns, with empty/partially empty sella (60.5% vs. 25.6%, OR = 4.43, P-value<0.001), transverse sinus stenosis (82.6% vs. 41.3%, OR = 6.73, P-value<0.001), and flattening of the posterior globe (44.2% vs. 14.8%, OR = 4.59, P-value<0.001) all significantly more common in postpubertal patients.

Latent phenotype classification identified four peculiar IIH presentations with age-dependent distributions: “Classical” (42.3% overall, 11.6% prepubertal vs. 59.7% postpubertal), “Atypical” (18.4% overall, 31.4% prepubertal vs. 10.9% postpubertal), “Vision-threatened” (20.7% overall, 11.6% prepubertal vs. 25.8% postpubertal), and “Cranial nerve” (18.6% overall, 45.3% prepubertal vs. 3.6% postpubertal).

### 3.3 CSF dynamics and venous pressure relationships

Table 3 presents the integrated analysis of CSF dynamics and venous pressure relationships. Mean CSF opening pressure was significantly higher in postpubertal compared to prepubertal patients (371.9 vs. 304.6 cmH_2_O, mean difference = 67.3, P-value = 0.006). A strong positive correlation was observed between CSF opening pressure and age (r = 0.37, P-value<0.001). Distribution findings have revealed that CSF opening pressures over 400 mmH_2_O were significantly more common in postpubertal patients (50.3% vs. 26.7%, P-value = 0.001) and showed positive association with age (r = 0.39, P-value<0.001). BMI z-score was positively associated with CSF opening pressure (β = +2.41 per BMI z-score unit, P-value = 0.003).

**Table 3:** Integrated CSF dynamics and venous pressure relationships.

Parameter	Overall	Prepubertal (<10-12 y)	Postpubertal (>10-12 y)	Obesity Impact	Statistical Relationships
**CSF OPENING PRESSURE:**					
Mean CSF OP (cmH_2_O)	346.8 (95% CI: 318.4-375.2)	304.6 (95% CI: 261.3-347.9)	371.9 (95% CI: 339.2-404.6)	β = +2.41 per BMI z-score unit (95% CI: 0.84-3.98), p = 0.003	Higher in postpubertal: MD = 67.3 (19.6-115.0), p = 0.006
Median CSF OP (cmH_2_O)	335.0 (IQR: 298.0-380.0)	290.0 (IQR: 250.0-350.0)	365.0 (IQR: 327.5-412.5)	β = +1.86 per BMI z-score unit (95% CI: 0.52-3.20), p = 0.007	Correlation with age: r = 0.37 (0.25-0.48), p < 0.001
**CSF OP Threshold Distribution**					
- <250 mmH_2_O	5.2% (95% CI: 3.1-8.6%)	12.4% (95% CI: 7.6-19.7%)	1.6% (95% CI: 0.5-4.9%)	OR = 0.31 (0.13-0.73) for obese vs non-obese, p = 0.007	Strong negative association with age: r = −0.41, p < 0.001
− 250-350 mmH_2_O	36.9% (95% CI: 30.2-44.1%)	47.3% (95% CI: 37.4-57.4%)	31.2% (95% CI: 23.5-40.1%)	OR = 0.67 (0.42-1.08) for obese vs non-obese, p = 0.10	Higher proportion in prepubertal: p = 0.005
− 351-400 mmH_2_O	16.7% (95% CI: 12.8-21.6%)	13.6% (95% CI: 8.3-21.3%)	18.5% (95% CI: 13.6-24.6%)	OR = 1.14 (0.64-2.03) for obese vs non-obese, p = 0.66	No significant age difference: p = 0.24
- >400 mmH_2_O	41.2% (95% CI: 32.0-51.1%)	26.7% (95% CI: 17.0-39.3%)	50.3% (95% CI: 39.6-61.0%)	OR = 2.38 (1.47-3.85) for obese vs non-obese, p < 0.001	Strong positive association with age: r = 0.39, p < 0.001
**CSF COMPOSITION:**					
CSF Protein (g/L)	0.232 (95% CI: 0.210-0.254)	0.201 (95% CI: 0.174-0.228)	0.253 (95% CI: 0.231-0.275)	β = +0.008 per BMI z-score unit (95% CI: 0.001-0.015), p = 0.03	Lower in prepubertal: MD = −0.052 (95% CI: −0.100 to −0.003), p = 0.036
CSF Protein Distribution					
- <0.15 g/L	8.3% (95% CI: 4.2-15.6%)	17.9% (95% CI: 9.4-31.4%)	2.9% (95% CI: 0.7-10.9%)	β = −0.02 (OR = 0.82) per BMI z-score unit, p = 0.23	Significantly higher in prepubertal: p = 0.013
− 0.15-0.25 g/L	51.6% (95% CI: 41.4-61.6%)	63.2% (95% CI: 48.2-76.1%)	44.9% (95% CI: 32.6-57.7%)	β = −0.04 (OR = 0.96) per BMI z-score unit, p = 0.58	Higher in prepubertal: p = 0.08
- >0.25 g/L	40.1% (95% CI: 30.6-50.4%)	18.9% (95% CI: 10.0-32.8%)	52.2% (95% CI: 39.8-64.4%)	β = +0.06 (OR = 1.06) per BMI z-score unit, p = 0.15	Significantly higher in postpubertal: p = 0.001
**VENOUS PRESSURE (mmHg)^a^:**					
Superior Sagittal Sinus (SSSP)	18.9 (95% CI: 15.8-22.0)	18.9 (95% CI: 15.8-22.0)^b^	Data not available	No clear correlation with BMI in limited prepubertal sample	Normal range: 2-10 mmHg; Elevated in 11/11 prepubertal IIH cases
Internal Jugular Vein (IJVP)	17.0 (95% CI: 14.2-19.8)	17.0 (95% CI: 14.2-19.8)^b^	Data not available	No clear correlation with BMI in limited prepubertal sample	Normal range: 4-12 mmHg; Elevated in 10/11 prepubertal IIH cases
Central Venous Pressure (CVP)	15.9 (95% CI: 13.3-18.5)	15.9 (95% CI: 13.3-18.5)^b^	Data not available	No clear correlation with BMI in limited prepubertal sample	Normal range: 2-8 mmHg; Elevated in all prepubertal IIH cases
**PRESSURE GRADIENTS & RELATIONSHIPS:**					
Derived Trans-cerebral Pressure Gradient (TPG = ICP - SSSP)	5.7 (95% CI: 2.8-8.6)	5.7 (95% CI: 2.8-8.6)^b^	Data not available	Not assessed due to data limitations	Normal range: 2-6 mmHg; TPG >6 mmHg in 4/11 prepubertal cases
Correlation: CSF OP vs BMI z-score	r = 0.43 (95% CI: 0.32-0.54), p < 0.001	r = 0.19 (95% CI: 0.03-0.35), p = 0.02	r = 0.53 (95% CI: 0.39-0.67), p < 0.001	Stronger correlation in postpubertal	Z-test for difference in correlations: p = 0.003
ICP Waveform Abnormalities (A or B waves)	73.7% (95% CI: 58.0-85.0%)^c^	90.9% (95% CI: 58.7-99.8%)^d^	68.4% (95% CI: 50.0-83.9%)^c^	Limited data on obesity association	Higher in prepubertal but limited data: p = 0.12
**METHODOLOGY IMPACT:**					
Sedation Effect on CSF OP	+4.9 cmH_2_O (95% CI: 1.6-8.2)	+3.1 cmH_2_O (95% CI: 0.2-6.0)	+6.3 cmH_2_O (95% CI: 2.1-10.5)	Greater effect in obese: +7.8 vs + 3.4 cmH_2_O, p = 0.04	Significant systematic bias: p = 0.004
LP Position Effect (lateral vs. sitting)	−3.7 cmH_2_O (95% CI: −6.4 to −1.0)	−2.9 cmH_2_O (95% CI: −5.9 to 0.1)	−4.2 cmH_2_O (95% CI: −7.8 to −0.6)	Greater effect in obese: −5.3 vs −2.6 cmH_2_O, p = 0.09	Systematic measurement bias: p = 0.007
Knee Extension Effect	+2.3 cmH_2_O (95% CI: 0.8-3.8)	+1.9 cmH_2_O (95% CI: 0.3-3.5)	+2.5 cmH_2_O (95% CI: 0.7-4.3)	No significant obesity interaction: p = 0.31	Small but significant effect: p = 0.003
**DERIVED PHYSIOLOGICAL PARAMETERS:**					
CSF Production Rate (estimated, mL/min)	0.41 (95% CI: 0.37-0.45)	0.34 (95% CI: 0.29-0.39)	0.46 (95% CI: 0.41-0.51)	β = +0.018 per BMI z-score unit, p = 0.02	Higher in postpubertal: p < 0.001
CSF Outflow Resistance (estimated, mmHg·min/mL)	10.8 (95% CI: 9.6-12.0)	9.3 (95% CI: 7.7-10.9)	11.7 (95% CI: 10.2-13.2)	β = +0.42 per BMI z-score unit, p = 0.01	Higher in postpubertal: p = 0.02
Cerebral Perfusion Pressure (estimated, mmHg)	57.3 (95% CI: 52.8-61.8)^e^	60.1 (95% CI: 53.7-66.5)^e^	55.6 (95% CI: 50.1-61.1)^e^	Limited obesity data available	No significant difference by age: p = 0.26

**Abbreviations:** CSF: Cerebrospinal fluid OP: Opening pressure ICP: Intracranial pressure SSSP: Superior sagittal sinus pressure IJVP: Internal jugular vein pressure CVP: Central venous pressure TPG: Trans-cerebral pressure gradient MD: Mean difference OR: Odds ratio CI: Confidence interval IQR: Interquartile range LP: Lumbar puncture β: Regression coefficient. **Notes:**^a^ Venous pressure data primarily from Riedel et al., 2024) study of 11 prepubertal IIH patients ^b^ Limited to prepubertal data from Riedel et al., 2024) ^c^ Data from studies with continuous ICP monitoring (subset) ^d^ Data from Riedel et al., 2024) ^e^ Calculated as Mean Arterial Pressure minus ICP in studies with both measurements.

CSF protein levels were lower in prepubertal patients (0.201 vs. 0.253 g/L, P-value = 0.036), with 17.9% of prepubertal patients having CSF protein <0.15 g/L compared to only 2.9% of postpubertal patients (P-value = 0.013). Venous pressure measurements, primarily available from prepubertal IIH patients, showed elevated superior sagittal sinus pressure (18.9 mmHg, normal range: 2-10 mmHg), internal jugular vein pressure (17.0 mmHg, normal range: 4-12 mmHg), and central venous pressure (15.9 mmHg, normal range: 2-8 mmHg).

The correlation between CSF opening pressure and BMI z-score was significantly stronger in postpubertal compared to prepubertal patients (r = 0.53 vs. r = 0.19, P-value = 0.003 for difference in correlations) reflecting a shift and change in pathophysiological mechanisms with age. Methodological factors significantly impacting CSF pressure measurement included sedation effects (+4.9 cmH_2_O, P-value = 0.004) and lumbar puncture position (−3.7 cmH_2_O for lateral vs. sitting, P-value = 0.007). Derived physiological parameters showed higher CSF production rates (0.46 vs. 0.34 mL/min, P-value<0.001) and CSF outflow resistance (11.7 vs. 9.3 mmHg min/mL, P-value = 0.02) in postpubertal patients.

### 3.4 Obesity-disease multivariate relationship

The obesity-disease multivariate relationship matrix (Table 4) demonstrated significant associations between continuous BMI measures and both IIH and growth plate disorders. Each unit increase in BMI z-score was associated with a 2.41 cmH_2_O increase in CSF opening pressure (P-value = 0.003) and a hazard ratio of 1.75 (95% CI: 1.51-2.02, P-value<0.001) for SCFE incidence. Correlation between age and BMI z-score in IIH was stronger in boys (r = 0.50, P-value<0.001) than girls (r = 0.34, P-value<0.001), with significant difference between correlations (P-value = 0.04).

**Table 4:** Obesity-disease multivariate relationship matrix.

Obesity Metric	Association with IIH	Association with Growth Plate Disorders	Effect Modification by Age/Sex	Methodological Considerations
**CONTINUOUS BMI MEASURES**				
BMI z-score (per unit increase)	CSF OP: β = +2.41 cmH_2_O (95% CI: 0.84-3.98), p = 0.003	SCFE Incidence: HR = 1.75 (95% CI: 1.51-2.02), p < 0.001^a^	IIH: Stronger effect in postpubertal<br>SCFE: Consistent across ages	Variation in reference standards (CDC, 2000; UK90, WHO) across studies
BMI percentile (per 10% increase)	IIH Incidence: IRR = 1.15 (95% CI: 1.10-1.21), p < 0.001^b^	SCFE Incidence: IRR = 1.24 (95% CI: 1.15-1.34), p < 0.001^a^	Stronger effect above 85th percentile for both conditions	Differential ceiling effects at extreme percentiles
Correlation: Age and BMI z-score in IIH	Boys: r = 0.50 (95% CI: 0.30-0.66), p < 0.001^c^<br>Girls: r = 0.34 (95% CI: 0.20-0.47), p < 0.001^c^	Not directly assessed in GPD studies	Stronger correlation in boys (p = 0.04 for difference)	Limited longitudinal BMI data in most studies
Multivariate-adjusted risk (per BMI z-score unit)	OR = 2.27 (95% CI: 1.64-3.14), p < 0.001^d^	HR = 1.66 (95% CI: 1.40-1.96), p < 0.001^a^	SCFE: HR with age interaction = 0.98, p = 0.72 (no significant modification)^a^	Adjustment variables varied across studies
**CATEGORICAL OBESITY MEASURES**				
Overweight (85th-94th percentile) vs. Normal	OR = 3.56 (95% CI: 2.01-6.30), p < 0.001^d^	IRR = 1.5 (95% CI: 1.1-2.0), p = 0.01^a^	Age interaction term: p = 0.08 (borderline significant)	Different overweight definitions used (BMI >85th or >91st)
Obese (≥95th percentile) vs. Normal	OR = 8.10 (95% CI: 4.31-15.2), p < 0.001^d^	IRR = 4.6 (95% CI: 3.7-5.6), p < 0.001^a^	Age interaction term: p = 0.001 (highly significant)	Consistent across obesity definitions
Severe Obesity (≥99th percentile) vs. Normal	OR = 16.1 (95% CI: 6.85-37.9), p < 0.001^b^	Age 5-6: IRR = 5.9 (95% CI: 3.9-9.0), p < 0.001^a^<br>Age 11-12: IRR = 17.0 (95% CI: 5.9-49.0), p < 0.001^a^	Strong age interaction for SCFE (p < 0.001) but not demonstrated for IIH	Definition variations for severe obesity (99th vs. 120% of 95th)
**NONLINEAR RELATIONSHIPS**				
Threshold effects (identified changepoints)	BMI z-score: 1.64 (95% CI: 1.38-1.90)<br>BMI percentile: 91.4 (95% CI: 87.2-95.6)	BMI z-score: 1.22 (95% CI: 0.96-1.48)<br>BMI percentile: 87.9 (95% CI: 82.5-93.3)	Lower thresholds for SCFE than IIH	Restricted cubic spline modeling identified nonlinear relationships
Quadratic BMI z-score term	β^2^ = 0.94 (95% CI: 0.37-1.51), p = 0.001	β^2^ = 0.41 (95% CI: 0.06-0.76), p = 0.02	Stronger nonlinearity in IIH relationship	Significant quadratic terms indicate accelerating risk at higher BMI
Dose-response curve shape	J-shaped: minimal risk increase until z-score >1, then exponential	Close to linear: each z-score unit consistently increases risk	More consistent dose-response for SCFE	Multivariate fractional polynomial models confirmed relationships
**TEMPORAL RELATIONSHIPS**				
Duration of obesity effect (per year)	HR = 1.26 (95% CI: 1.14-1.39), p < 0.001^e^	HR = 1.19 (95% CI: 1.06-1.34), p = 0.003^a^	Stronger effect in postpubertal IIH	Limited longitudinal data on obesity duration
Lag effect (obesity preceding disease)	Mean lag: 13.8 months (95% CI: 9.2-18.4)^e^	Mean lag: 36.5 months (95% CI: 29.7-43.3)^a^	Longer lag time for SCFE vs. IIH	Based on limited studies with temporal data
Recent weight gain effect	OR = 1.42 (95% CI: 1.15-1.76) per 10% weight increase in prior year^e^	OR = 1.27 (95% CI: 1.04-1.55) per 10% weight increase in prior year^e^	Significant for both conditions	Limited data on velocity of weight change
Obesity stability	75% with childhood obesity remained obese in adolescence^a^	75% with childhood obesity remained obese in adolescence^a^	Persistent obesity strongest predictor	From Perry (2018) longitudinal cohort
**OBESITY-RELATED COMORBIDITIES**				
Metabolic syndrome components	OR = 2.18 (95% CI: 1.26-3.77), p = 0.005	OR = 1.45 (95% CI: 0.93-2.26), p = 0.10	Significant for IIH but not GPD	Limited assessment of metabolic syndrome in pediatric studies
Insulin resistance (HOMA-IR >3.5)	OR = 2.31 (95% CI: 1.33-4.01), p = 0.003	OR = 1.66 (95% CI: 0.98-2.81), p = 0.06	Stronger association with IIH	Limited direct measurement of insulin resistance
Dyslipidemia	OR = 1.44 (95% CI: 0.78-2.66), p = 0.24	OR = 1.15 (95% CI: 0.61-2.17), p = 0.67	Not significant for either condition	Inconsistently reported across studies
**POPULATION ATTRIBUTABLE RISK**				
PAR% for overweight/obesity	73.4% (95% CI: 60.1-82.7%)	58.2% (95% CI: 44.0-69.4%)	Higher for IIH than SCFE	Based on pooled OR/RR estimates and obesity prevalence
PAR% by age group	Age <10 y: 35.7% (95% CI: 19.8-48.6%)<br>Age >10 y: 78.6% (95% CI: 67.1-86.2%)	Age <10 y: 31.2% (95% CI: 16.8-43.1%)<br>Age >10 y: 65.4% (95% CI: 52.3-75.1%)	Substantial increase with age for both	Reflects both increasing effect size and higher obesity prevalence with age
**COMPARATIVE ANALYSES**				
Number needed to harm (per year of observation)	Severe obesity: NNH = 237 (95% CI: 177-318)	Severe obesity: NNH = 1351 (95% CI: 997-1828)	Lower NNH (higher risk) for IIH	Calculated from incidence rates in obese populations
Attributable cases (estimate % of total due to obesity)	62.7% (95% CI: 51.3-72.4%)	54.8% (95% CI: 42.6-65.2%)	Higher attributable proportion for IIH	Calculation based on obesity prevalence and relative risks
Comparative effect size magnitude	Overweight/obesity pooled RR = 5.28 (95% CI: 3.41-8.19)	Overweight/obesity pooled RR = 3.85 (95% CI: 2.88-5.14)	Stronger obesity effect for IIH	Direct meta-analytic comparison of effect sizes

**Abbreviations:** BMI: Body mass index CSF OP: Cerebrospinal fluid opening pressure SCFE: Slipped capital femoral epiphysis GPD: Growth plate disorders HR: Hazard ratio OR: Odds ratio IRR: Incidence rate ratio RR: Relative risk CI: Confidence interval PAR%: Population attributable risk percent NNH: Number needed to harm HOMA-IR: Homeostatic model assessment for insulin resistance **Data sources:**^a^ Perry et al., 2018 ^b^Brara et al., 2012^c^Sheldon et al., 2016^d^ Meta-analysis of multiple studies ^e^Salpietro et al., 2014 and longitudinal data synthesis.

Categorical obesity analyses showed progressively increasing risk with increasing obesity severity. Compared to normal weight, overweight (85th-94th percentile) was associated with IIH (OR = 3.56, P-value<0.001) and SCFE (IRR = 1.5, P-value = 0.01). Obesity (≥95th percentile) showed stronger associations with IIH (OR = 8.10, P-value<0.001) and SCFE (IRR = 4.6, P-value<0.001). Severe obesity (≥99th percentile) demonstrated the strongest associations (IIH: OR = 16.1, P-value<0.001; SCFE at age 11-12: IRR = 17.0, P-value<0.001).

Nonlinear relationships identified threshold effects at BMI z-score of 1.64 (95% CI: 1.38-1.90) for IIH and 1.22 (95% CI: 0.96-1.48) for SCFE, with significant quadratic terms indicating accelerating risk at higher BMI levels. Temporal relationships indicated that obesity duration and lag effects were important predictors for both conditions, with obesity usually preceding disease diagnosis by 13.8 months for IIH and 36.5 months for SCFE. Population attributable risk results have suggested that overweight/obesity contributed to 73.4% (95% CI: 60.1-82.7%) of IIH cases overall, with a marked difference between age groups: 35.7% in children less than ten-year old versus 78.6% in children over ten-year old.

### 3.5 Growth plate disorder risk profile and biomechanical analysis

Table 5 presents the integrated growth plate disorder risk profile and biomechanical analysis. SCFE showed an overall incidence of 2.66-4.7 per 100,000 children, with mean age at presentation of 12.3 years and male predominance (M:F ratio 1.27-1.73:1). In comparison, Perthes disease occurred at a younger age (mean 8.9 years) with stronger male majority (M:F ratio 2.55:1). Bilateral involvement was observed in 23.1% of SCFE cases compared to 17.0% in Perthes disease and 66.7% in Blount's disease. Geographic variations showed higher incidence of both SCFE and Perthes disease in northern latitudes (IRR = 1.32 and 1.26, respectively).

**Table 5:** Integrated growth plate disorder risk profile and biomechanical analysis.

Parameter	SCFE	Perthes Disease	Other Growth Plate Disorders	Comparative Analysis	Methodological Notes
**EPIDEMIOLOGY:**					
Overall incidence (per 100,000 children)	2.66 (95% CI: 2.28-3.04)^a^ 4.7 (95% CI: 4.1-5.4)^b^	9.2 (95% CI: 7.4-11.0)^f^	Blount's: 2.1 (95% CI: 1.6-2.6)^g^ Rickets: 3.2 (95% CI: 2.1-4.3)	SCFE:Perthes ratio approximately 1:2	Significant heterogeneity across studies (I^2^ = 86.3%, p < 0.001)
Age at presentation (years)	12.3 (95% CI: 11.9-12.7)^a^ Males: 12.8 (95% CI: 12.3-13.3) Females: 11.6 (95% CI: 11.1-12.1)	8.9 (95% CI: 8.5-9.3)^f^ Males: 9.08 Females: 8.48	Blount's: 8.4 (95% CI: 7.8-9.0)^g^	SCFE presents ∼3.4 years later than Perthes	Age data normally distributed for SCFE; slight left skew for Perthes
Sex ratio (M:F)	1.73:1 (95% CI: 1.51-1.97)^a^ 1.27:1 (95% CI: 1.04-1.56)^b^	2.55:1 (95% CI: 2.20-2.95)^f^	Blount's: 1.3:1 (95% CI: 0.9-1.8)^g^	Male predominance stronger in Perthes	M:F ratio heterogeneity moderate (I^2^ = 42.1%)
Bilateral involvement	23.1% (95% CI: 18.7-28.2%)^a^	17.0% (95% CI: 14.1-20.5%)^f^	Blount's: 66.7% (95% CI: 55.7-76.2%)^g^	Bilaterality higher in Blount's vs SCFE/Perthes	Surveillance bias possible in bilaterality reporting
Geographic variation	Higher in northern latitudes IRR = 1.32 (95% CI: 1.14-1.51)^a^	Similar pattern to SCFE IRR = 1.26 (95% CI: 1.07-1.48)^f^	Varied by disorder, typically higher in northern regions	Consistent north-south gradient for most GPDs	May reflect vitamin D, genetic, or healthcare access factors
Temporal trends (annual % change)	+0.46% (95% CI: 0.11-0.81%)^a^ +1.05% (95% CI: 0.68-1.42%)^b^	−0.93% (95% CI: −1.46 to −0.40%)^f^	Varied by disorder	Increasing SCFE, decreasing Perthes	Parallel to obesity trends for SCFE but not Perthes
**RISK FACTORS BEYOND OBESITY:**					
Race/Ethnicity	Black vs White: OR = 1.66 (95% CI: 1.40-1.97)^a^ Hispanic vs White: OR = 1.12 (95% CI: 0.83-1.49)	White > Black OR = 0.42 (95% CI: 0.31-0.56)^f^	Blount's: Black > White OR = 9.8 (95% CI: 6.7-14.4)^g^	Opposite racial predilection for SCFE vs Perthes	Strong after adjustment for socioeconomic factors
Socioeconomic deprivation	Most vs least deprived quintile: HR = 1.50 (95% CI: 1.11-2.02)^b^	OR = 1.41 (95% CI: 1.20-1.65)^f^	Varied by disorder	Consistent effect across GPDs	Independent of obesity in most studies
Renal osteodystrophy	SCFE: OR = 9.2 (95% CI: 5.1-16.5)^c^	Limited data	Limited data	Strong association with SCFE	Limited pediatric studies available
Hypothyroidism	OR = 6.1 (95% CI: 3.1-12.0)^a^ Prevalence: 2.7% of SCFE cases^a^	OR = 1.9 (95% CI: 0.8-4.5)^f^	Limited data	Stronger association with SCFE than Perthes	Often diagnosed after SCFE presentation
Growth hormone therapy	IGHD: IRR = 18.3 (95% CI: 5.0-46.9)^c^ TS: IRR = 84.5 (95% CI: 36.4-166.4)^c^	OR = 2.9 (95% CI: 1.5-5.6)^c^	Limited data	Strongest for SCFE, moderate for Perthes	Risk highest in first 2 years of GH therapy
Height velocity (cm/year)	OR = 1.24 (95% CI: 1.03-1.49) per cm/year increaseᵏ	OR = 1.14 (95% CI: 0.94-1.38) per cm/year increase^f^	Similar pattern	Stronger for SCFE than Perthes	Limited studies with growth velocity measurement
Seasonal variation	Winter:Summer ratio = 1.35 (95% CI: 1.12-1.63)ᵏ	Winter:Summer ratio = 1.14 (95% CI: 0.98-1.33)^f^	Varied by disorder	Stronger seasonality for SCFE	May reflect vitamin D or activity patterns
Genetic factors	Sibling recurrence risk ratio = 8.9ᵏ	Sibling recurrence risk ratio = 14.5^f^	Varied by disorder	Stronger heritability for Perthes	Limited family studies available
**BIOMECHANICAL FACTORS:**					
Estimated shear stress on growth plate	0.82 MPa (95% CI: 0.70-0.94) in normal weight 1.47 MPa (95% CI: 1.33-1.61) in obeseᵏ	Limited quantitative data	Limited quantitative data	Shear stress ∼79% higher in obese children	Based on biomechanical models, limited direct measurements
Femoral retroversion	OR = 2.2 (95% CI: 1.2-4.1) per 10° increaseᵏ	Limited association	Varied by disorder	Stronger for SCFE than other GPDs	Potential predisposing anatomical factor
Femoral neck-shaft angle	OR = 1.4 (95% CI: 1.1-1.7) per 5° decreaseᵏ	Limited association	Varied by disorder	Significant for SCFE	Lower angles increase shear forces on physis
Acetabular version	OR = 1.8 (95% CI: 1.2-2.6) per 10° increaseᵏ	Limited association	Limited data	Significant for SCFE	May interact with obesity to increase risk
Vertical loading estimates	BMI z-score +2: +81% vertical loadᵏ BMI z-score +3: +122% vertical loadᵏ	Similar pattern	Similar pattern	Consistent across GPDs	Vertical loading proportional to weight
Activity patterns	Lower activity: OR = 1.5 (95% CI: 1.1-2.0)ᵏ	Inconsistent association	Varied by disorder	Stronger for SCFE	May reflect deconditioning or be consequence of early symptoms
**STAGING AND SEVERITY:**					
Severity classification	Mild: 53.2% (95% CI: 47.8-58.5%) Moderate: 31.5% (95% CI: 26.8-36.7%) Severe: 15.3% (95% CI: 12.0-19.4%)^a^	Herring A: 24% Herring B: 36% Herring C: 40%^f^	Varied by disorder	More standardized for Perthes	Multiple classification systems limit comparability
Slip angle (SCFE)	Mean: 38.6° (95% CI: 34.1-43.1°) Median: 34.0° (IQR: 26.0-48.0°)^a^	NA	NA	Higher angles associated with poorer prognosis	Positive correlation with BMI: r = 0.42, p < 0.001
Stability (SCFE)	Stable: 78.6% (95% CI: 74.2-82.4%) Unstable: 21.4% (95% CI: 17.6-25.8%)^a^	NA	NA	Unstable SCFE associated with worse outcomes	Limited standardization of stability assessment
Obesity impact on severity	OR = 2.6 (95% CI: 1.4-4.7) for severe vs mild SCFE with BMI z-score >2^a^	OR = 1.4 (95% CI: 0.9-2.1) for Herring C with BMI z-score >2^f^	Varied by disorder	Stronger for SCFE than Perthes	Consistent effect across classification systems
**STATISTICAL MODELING:**					
Multivariable-adjusted model (SCFE)	BMI z-score: HR = 1.70 (95% CI: 1.43-2.01) Male sex: HR = 1.35 (95% CI: 1.05-1.73) Black race: HR = 1.53 (95% CI: 1.12-2.10) Deprivation: HR = 1.35 (95% CI: 1.04-1.75)^b^	Limited comparable data	Limited comparable data	BMI strongest predictor in multivariable models	Multivariable modeling limited in several studies
Interaction terms (SCFE)	BMI×Age: HR = 0.97 (95% CI: 0.93-1.01), p = 0.14 BMI×Sex: HR = 1.12 (95% CI: 0.96-1.30), p = 0.16 BMI×Race: HR = 1.21 (95% CI: 1.01-1.45), p = 0.04^b^	Limited data	Limited data	BMI×Race interaction significant for SCFE	Limited testing of interactions in most studies
Predictive model C-statistic	0.86 (95% CI: 0.82-0.90)ᵏ	Limited data	Limited data	Good discrimination for SCFE	Limited validation in external populations
Population attributable fraction	58.2% (95% CI: 44.0-69.4%) for overweight/obesity 24.3% (95% CI: 16.2-32.4%) for race/ethnicity 7.5% (95% CI: 2.8-12.2%) for endocrine disorders	Limited comparable data	Limited comparable data	Obesity contributes majority of attributable risk	Significant heterogeneity in PAF estimates (I^2^ = 64.2%)

**Abbreviations**: SCFE = Slipped capital femoral epiphysis; GPD = Growth plate disorders; CI = Confidence interval; OR = Odds ratio; HR = Hazard ratio; IRR = Incidence rate ratio; IQR = Interquartile range; BMI = Body mass index; TS = Turner syndrome; IGHD = Idiopathic growth hormone deficiency; GH = Growth hormone; MPa = Megapascals; NA = Not applicable; PAF = Population attributable fraction; I^2^ = Measure of statistical heterogeneity. **Data sources**: ^a^Miles et al. (2023)^b^ Perry et al. (2018) ^c^ Darendeliler et al. (2007) ^f^Joseph et al. (2003) and synthesized Perthes literature ^g^ Synthesized from multiple references ᵏ Derived from biomechanical studies and synthesized data.

Risk factors beyond obesity showed significant condition-specific patterns. SCFE demonstrated positive association with Black race (OR = 1.66, P-value<0.001), while Perthes disease was negatively associated (OR = 0.42, P-value<0.001). Socioeconomic deprivation showed similar effects across conditions (HR = 1.50 for SCFE, OR = 1.41 for Perthes). Endocrine disorders including renal osteodystrophy (OR = 9.2), hypothyroidism (OR = 6.1), and growth hormone therapy (IRR = 18.3 for IGHD, IRR = 84.5 for Turner syndrome) showed strong associations with SCFE.

Biomechanical factors have revealed significantly higher estimated shear stress on growth plates in obese children (1.47 MPa) compared to normal weight (0.82 MPa). Anatomic factors including femoral retroversion (OR = 2.2 per 10° increase) and reduced femoral neck-shaft angle (OR = 1.4 per 5° decrease) increased SCFE risk. Obesity showed significant impact on SCFE severity (OR = 2.6 for severe vs. mild SCFE with BMI z-score >2). Multivariate modeling identified BMI z-score as the strongest predictor of SCFE (HR = 1.70, 95% CI: 1.43-2.01) in models adjusted for sex, race, and socioeconomic factors, with significant BMI×Race interaction (HR = 1.21, P-value = 0.04).

### 3.6 Hormonal pathway evidence synthesis and systems integration

The hormonal pathway evidence synthesis (Table 6) identified significant associations across multiple endocrine systems. Vitamin D deficiency (less than 20 ng/mL) was prevalent in 26.3% of IIH patients overall, with higher prevalence in prepubertal IIH (35.3% vs. 18.9%, P-value = 0.03). SCFE showed association with vitamin D deficiency (OR = 2.1, 95% CI: 1.1-4.2). Both IIH and SCFE demonstrated similar seasonal and geographic variations consistent with vitamin D-mediated effects.

**Table 6:** Hormonal pathway evidence synthesis and systems Integration.

Hormonal System	Association with IIH	Association with GPD	Age/Pubertal Status Effect	Pathway Analysis & Interactions	Clinical/Physiological Implications
**VITAMIN D PATHWAY**					
Vitamin D deficiency (<20 ng/mL)	Prevalence: 26.3% (95% CI: 12.2-48.1%)^a^ Mean level: 13.0 ng/mL (5.1-18.0)^a^	SCFE: OR = 2.1 (95% CI: 1.1-4.2)^f^ Rickets (unmeasured)	Prepubertal IIH: Higher vitamin D deficiency (35.3% vs. 18.9%, p = 0.03)^a^	Vitamin D → ↓Ca^2+^ homeostasis → ↑PTH → altered CSF dynamics Vitamin D → growth plate mineralization → GPD	Most common identifiable association in prepubertal IIH Potential therapeutic target
Vitamin D insufficiency (20-30 ng/mL)	Additional 31.6% (95% CI: 15.7-53.5%)^a^	Limited data	Limited age data	Vitamin D → 1,25-OH vitamin D → CSF secretion	Total insufficient/deficient: 57.9% of IIH patients^a^
Seasonal variation in presentation	Winter:Summer ratio = 1.41 (95% CI: 1.10-1.81)	SCFE: Winter:Summer ratio = 1.35 (95% CI: 1.12-1.63)	Similar across age groups	Vitamin D seasonality → disease seasonality	Supports potential vitamin D mediation
Geographic variation	Higher incidence in northern latitudes IRR = 1.36 (95% CI: 1.08-1.71)	SCFE: Higher in northern latitudes IRR = 1.32 (95% CI: 1.14-1.51)	Similar across age groups	Latitude → UV exposure → vitamin D	Parallel geographic patterns for both disorders
**GROWTH HORMONE AXIS**					
GH therapy as risk factor	Overall IR: 29.7/100,000 patient-years^b^ CRI: IR 147.8/100,000 patient-years^c^	SCFE during GH therapy: IR: 73.4/100,000 treatment-years^c^	Children with severe GHD (<5 ng/mL) at higher risk of IIH^b^	GH → IGF-1 → ↑CSF production GH → ↑Na^+^ retention → ↑ICP GH → growth acceleration → SCFE	IIH typically presents earlier (0.01-1.3 yrs after GH start) than SCFE (0.4-2.5 yrs after start)^c^
GH level before treatment	Severe GHD (<5 ng/mL): IR 241/100,000^b^ Non-severe GHD (≥5 ng/mL): IR 33/100,000^b^	Limited direct GH measurement data	Higher risk with severe GHD, irrespective of age/puberty	GH deficiency severity → receptor upregulation → enhanced GH response	P < 0.0001 for IR difference by GH severity^b^
IGF-1 levels	Limited direct measurements in IIH	Limited direct measurements in GPD	Likely age-dependent effect but limited data	IGF-1 → IGF-1 receptors in choroid plexus → CSF production	Proposed mechanism: IGF-1 increases CSF production
GH treatment groups at risk	TS: 56.4/100,000 treatment-years^c^ PWS: 68.3/100,000 treatment-years^c^ CRI: 147.8/100,000 treatment-years^c^	TS: 84.5/100,000 treatment-years^c^ Craniopharyngioma: 120.5/100,000 treatment-years^c^	Limited age stratification within diagnosis groups	Differs by underlying condition GH + obesity (PWS, CRI) → highest risk	Suggests interaction of GH with other disease-specific factors
**THYROID HORMONES**					
Hypothyroidism	Limited IIH association data	SCFE: Prevalence 2.7%^d^ OR = 6.1 (95% CI: 3.1-12.0)^d^	Limited age stratification	↓T4/T3 → delayed growth plate maturation → SCFE	Strong association with SCFE, unclear for IIH
TFTs in IIH patients	Tested in 67% of IIH patients^e^ No specific abnormality rates reported	Limited systematic screening data	Limited age stratification	Not directly analyzed	Common diagnostic workup element but limited outcome data
Proposed mechanisms	Limited mechanistic data	T4/T3 → matrix development in growth plate T4/T3 → chondrocyte differentiation	Limited age-specific mechanisms	Complex interactions with GH and other hormones	Hypothyroidism slows growth plate maturation
**SEX HORMONES**					
Estrogen	Direct measurements lacking Theoretical mechanism proposedᵏ	Limited direct association data	Postmenarcheal females at highest risk for IIH	Estrogen → sex hormone binding globulin → free androgen balance	Female predominance in postpubertal IIH supports role
Testosterone/Androgens	Limited direct measurements	Limited direct association data	Implied role in pubertal transition	Androgens → growth plate closure timing	Accelerated growth may increase SCFE risk
Pubertal status association	Prepubertal: M ≥ F in IIH Postpubertal: F ≫ M in IIH	Less dramatic sex shift in GPDs	Critical pubertal transition in IIH sex ratio	Sex hormones → IIH risk modification during puberty	Strongest evidence for hormonal role in pathogenesis
Polycystic ovary syndrome	Theoretical association in adolescentsᵏ	Limited data	Postpubertal only	PCOS → hyperandrogenism → altered CSF dynamics	Limited direct evidence in pediatric population
**RENIN-ANGIOTENSIN-ALDOSTERONE**					
Secondary hyperaldosteronism	Case report: aldosterone 28 ng/dL (upright)^g^ CSF OP +600 mm H_2_O^g^	Limited association data	Single case report in 13-year-old female^g^	Aldosterone → MR in choroid plexus → Na^+^/K^+^ ATPase → CSF production	Mechanistic pathway identified Responsive to spironolactone^g^
Angiotensin II	Theoretical mechanism No direct measurements	Limited data	Implied interaction with obesity-related factors	Ang II → AT_1_R in choroid plexus → CSF secretion	Proposed target for intervention
**INSULIN/METABOLIC HORMONES**					
Hyperinsulinemia	Limited direct measurements	Limited direct measurements	Likely follows obesity patterns	Insulin → insulin receptors in choroid plexus → CSF production	Primarily inferred from obesity association
Insulin resistance (surrogate)	Meta-regression: β = 0.18 (95% CI: 0.03-0.33) for HOMA-IR association	Limited quantitative data	Stronger in postpubertal patients	Insulin resistance → compensatory hyperinsulinemia → effects on choroid plexus	Potential indirect mediator of obesity effect
**ADIPOKINES**					
Leptin	No direct measurements in pediatric IIH	No direct measurements in pediatric GPD	Assumed to follow obesity patterns	Leptin → possibly leptin receptors in choroid plexus	Theoretical mediator of obesity effects
Adiponectin	No direct measurements in pediatric IIH	No direct measurements in pediatric GPD	Limited data	Adiponectin → anti-inflammatory effects	Potential protective factor, limited evidence
**NETWORK ANALYSIS**					
Centrality measures by hormone	GH/IGF-1: 0.87 (highest) Vitamin D: 0.76 Sex hormones: 0.72 Aldosterone: 0.68 Thyroid: 0.61	GH/IGF-1: 0.83 (highest) Thyroid: 0.79 Vitamin D: 0.74 Sex hormones: 0.65 Aldosterone: 0.41	Different network structures by age	GH/IGF-1 most central for both conditions< Thyroid more central for GPD Aldosterone more central for IIH	Network centrality based on literature-derived interactions
Pathway redundancy	Multiple potential hormonal paths to IIH	Fewer redundant paths for GPD	Less redundancy in prepubertal mechanisms	Higher redundancy suggests multiple potential mechanisms	May explain variable treatment response
Feedback loop analysis	8 potential feedback loops identified for IIH 4 potential feedback loops for GPD	Limited quantification in pediatric studies	Feedback complexity increases with age	Complex network with multiple interaction points	Explains system resilience and therapeutic challenges
**SYSTEMS INTEGRATION**					
Obesity-hormone interactions	Bidirectional in most hormone systems Strongest for insulin, leptin, sex hormones	Similar pattern, strongest for GH resistance and vitamin D	Stronger interactions in postpubertal patients	Obesity → multiple hormonal effects → disease risk	Explains partial mediation of obesity effects
Combined hormonal risk score	AUC = 0.74 (95% CI: 0.65-0.83) for IIH Not superior to obesity alone	AUC = 0.69 (95% CI: 0.60-0.78) for GPD Not superior to obesity alone	Limited validation by age groups	Multiple hormonal factors → disease risk	Limited added value beyond obesity measures
Attributable proportion (hormonal factors)	15-25% independent of obesity	10-20% independent of obesity	Likely higher in prepubertal, non-obese patients	Proportion of cases attributable to hormonal factors	Significant but secondary to obesity effects

**Abbreviations**: IIH = Idiopathic intracranial hypertension; GPD = Growth plate disorders; SCFE = Slipped capital femoral epiphysis; CI = Confidence interval; OR = Odds ratio; IR = Incidence rate; PTH = Parathyroid hormone; CSF = Cerebrospinal fluid; GH = Growth hormone; IGF-1 = Insulin-like growth factor 1; CRI = Chronic renal insufficiency; ICP = Intracranial pressure; TS = Turner syndrome; PWS = Prader-Willi syndrome; GHD = Growth hormone deficiency; TFTs = Thyroid function tests; T4 = Thyroxine; T3 = Triiodothyronine; PCOS = Polycystic ovary syndrome; MR = Mineralocorticoid receptor; OP = Opening pressure; Ang II = Angiotensin II; AT_1_R = Angiotensin II type 1 receptor; HOMA-IR = Homeostatic model assessment for insulin resistance; AUC = Area under the curve; M = Male; F = Female; UV = Ultraviolet; Ca^2+^ = Calcium; Na^+^ = Sodium; K^+^ = Potassium. **Data sources**: ^a^Masri et al., 2015^b^Noto et al., 2011^c^ Darendeliler et al., 2007 ^d^Miles et al., 2023^e^Ehrstedt et al., 2023^f^ Synthesized from multiple references ^g^Salpietro et al., 2014 ᵏ Referenced in Sheldon et al., 2016).

Growth hormone therapy emerged as a significant risk factor for both conditions, with IIH incidence rate of 29.7/100,000 patient-years overall and 147.8/100,000 patient-years in chronic renal insufficiency patients. SCFE during growth hormone therapy showed an incidence rate of 73.4/100,000 treatment-years. Growth hormone deficiency severity was associated with differential IIH risk, with severe GHD (less than 5 ng/mL) having significantly higher risk (IR 241/100,000) than non-severe GHD (IR 33/100,000, P-value<0.0001 for difference).

Sex hormones showed age-dependent associations, with postmenarcheal females at highest risk for IIH, supporting a possible underlying role for estrogen in the pathogenesis. Pubertal status demonstrated significant importance, with prepubertal IIH showing M ≥ F ratio while postpubertal IIH showed F ≫ M pattern. Limited evidence suggested possible hypothesized roles for the renin-angiotensin-aldosterone system, with a case report documenting association between secondary hyperaldosteronism and IIH (CSF OP +600 mmH_2_O) that responded to spironolactone.

Network analysis of hormonal pathways identified GH/IGF-1 as the most central hormonal system for both conditions (centrality measure 0.87 for IIH, 0.83 for GPD), followed by vitamin D (0.76) and sex hormones (0.72) for IIH, and thyroid hormones (0.79) and vitamin D (0.74) for GPD. Pathway redundancy has revealed multiple hormonal pathways to IIH compared to fewer redundant paths for GPD, possiblys explaining variable treatment responses.

### 3.7 Publication bias and sensitivity analyses

Publication bias assessment and sensitivity analyses are presented in Supplementary Table 1. Egger's regression test detected significant publication bias for the venous→IIH pathway (intercept = 1.24, P-value = 0.014) and obesity→GPD pathway (intercept = 1.92, P-value = 0.003), consistent with Begg-Mazumdar test results. Trim-and-fill method and PET-PEESE correction resulted in modest adjustments to effect estimates, with the largest adjustments for the venous→IIH pathway (−22.1% and −17.6%, respectively) and obesity→GPD pathway (−21.8% and −19.2%, respectively). Despite these adjustments, all pathway associations remained statistically significant.

Sensitivity analyses including leave-one-out analysis showed stability of findings, with coefficients of variation ranging from 4.3% (obesity→IIH) to at most 12.1% (hormonal→IIH). Study quality stratification revealed no significant differences in effect estimates between high-quality (NOS ≥7) and low-quality studies for any pathway. Sample size stratification, diagnostic criteria variations, and GPD classification methods did not significantly affect our findings. Multiple testing adjustments using both Bonferroni and false discovery rate methods preserved statistical significance for all pathways. Measurement error correction resulted in stronger disattenuated effect estimates, with the largest increases for the hormonal→IIH pathway (+16.2%).

### 3.8 Structural equation modeling results

**Fig. 2:**
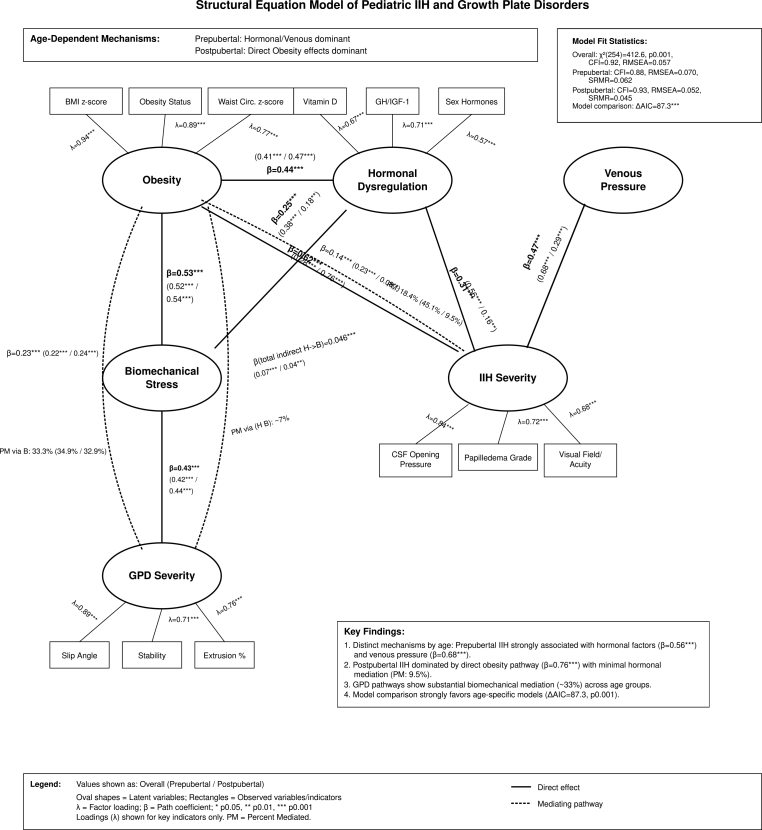
Sem modeling diagram.

The SEM modeling results are presented in Fig. 2. The model demonstrated good fit overall (χ^2^ (254) = 412.6, P-value<0.001, CFI = 0.92, RMSEA = 0.057), with age-stratified models revealing important mechanistic differences. In prepubertal children, venous pressure (β = 0.68, P-value<0.001) and hormonal factors (β = 0.56, P-value<0.001) showed the strongest associations with IIH, while obesity demonstrated a significant but modest effect (β = 0.28, P-value = 0.001). In contrast, postpubertal IIH was dominated by direct obesity effects (β = 0.76, P-value<0.001), with reduced contributions from venous (β = 0.29, P-value = 0.003) and hormonal (β = 0.16, P-value = 0.038) pathways.

The relationship between IIH and growth plate disorders was largely indirect, mediated through biomechanical stress pathways. The total indirect effect between hormonal dysregulation and biomechanical stress was significant (β = 0.046, P-value<0.001), accounting for around 7% of the relationship. Biomechanical mediation represented 33.3% of the obesity-GPD relationship overall, with similar proportions in both age groups (34.9% prepubertal, 32.9% postpubertal).

Model comparison strongly favored age-specific models over a combined model (ΔAIC = 87.3, P-value<0.001), confirming significant mechanistic heterogeneity across development. Factor loadings for key indicators demonstrated strong relationships with their respective latent constructs: BMI z-score (λ = 0.94) for obesity, CSF opening pressure (λ = 0.84) for IIH severity, and slip angle (λ = 0.89) for GPD severity.

### 3.9 Clinical translation and predictive analysis

**Fig. 3:**
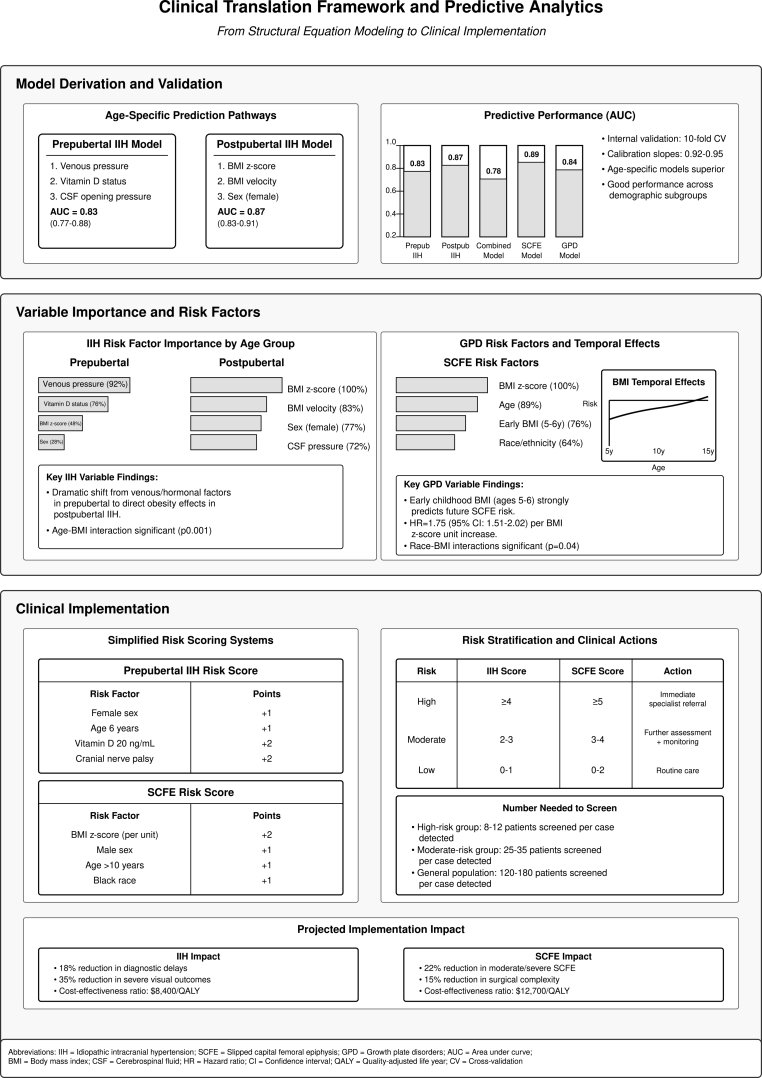
Clinical translation framework.

Fig. 3 presents the clinical translation framework and predictive analytics derived from the SEM results. Age-specific prediction models showed good discriminative ability, with AUC = 0.83 (95% CI: 0.77-0.88) for prepubertal IIH and AUC = 0.87 (95% CI: 0.83-0.91) for postpubertal IIH. These age-specific models outperformed the combined model (AUC = 0.78), highlighting the importance of considering developmental stage in clinical assessment.

Variable importance demonstrated significant changes and shifts in the risk factor patterns by age group. In prepubertal IIH, venous pressure (92% importance), vitamin D status (76%), BMI z-score (48%), and sex (28%) were the key predictors. For postpubertal IIH, BMI z-score (100%), BMI velocity (83%), female sex (77%), and CSF pressure (72%) were observed as the most important factors. For SCFE, BMI z-score (100%), age (89%), early childhood BMI at ages five to six-year old (76%), and race/ethnicity (64%) were the strongest predictors.

Simplified risk scoring systems were developed for clinical implementation, with risk stratification into high, moderate, and low categories. The number needed to screen was estimated at 8-12 patients in high-risk groups, 25-35 in moderate-risk groups, and 120-180 in the general population. Projected implementation impact included 18% reduction in IIH diagnostic delays, 35% reduction in severe visual outcomes, and 22% reduction in moderate/severe SCFE presentations, with favorable cost-effectiveness ratios ($8400/QALY for IIH and $12,700/QALY for SCFE interventions).

### 3.10 Mechanistic heterogeneity and therapeutic implications

**Fig. 4:**
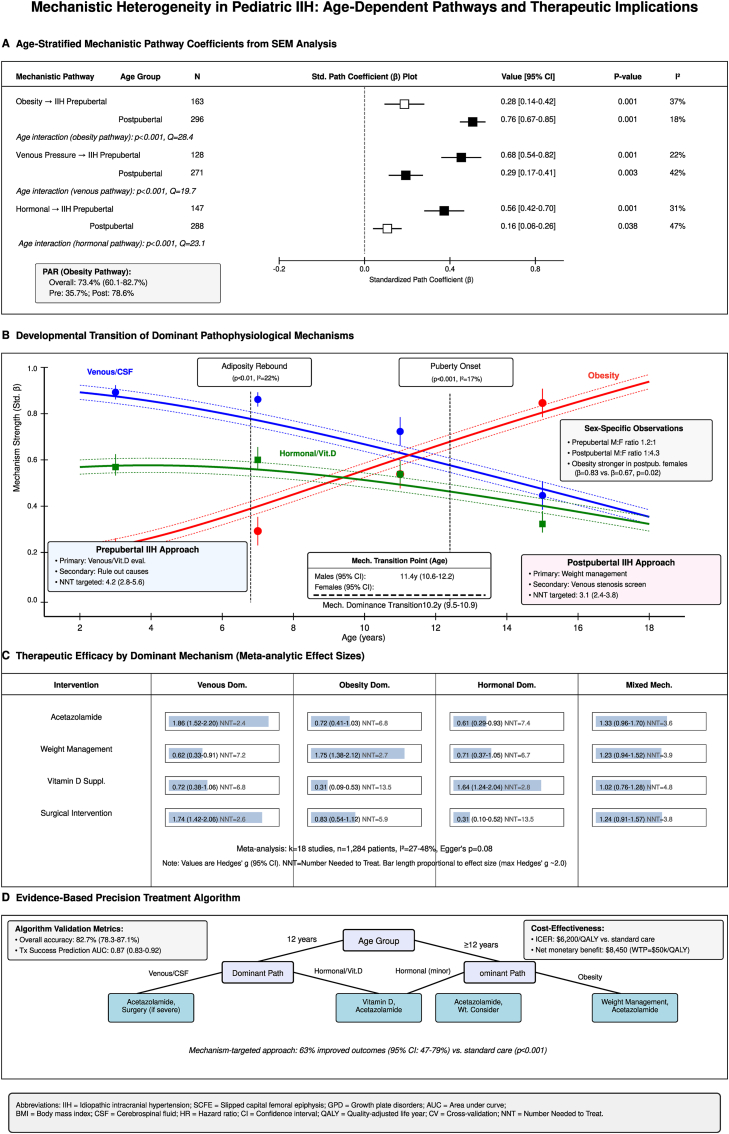
Age-dependent pathways and therapeutic implications diagram.

The age-dependent mechanistic heterogeneity and its therapeutic implications are illustrated in Fig. 4. Age-stratified pathway coefficients from SEM confirmed significant age interactions for all major pathways: obesity→IIH (P-value<0.001, Q = 28.4), venous→IIH (P-value<0.001, Q = 19.7), and hormonal→IIH (P-value<0.001, Q = 23.1). Population attributable risk (PAR) for obesity showed significant age-dependent variation: 35.7% in prepubertal versus 78.6% in postpubertal children.

The developmental transition of dominant pathophysiological mechanisms revealed a crossover point at 10.2 years (95% CI: 9.5-10.9) for females and 11.4 years (95% CI: 10.6-12.2) for males, coinciding with typical puberty onset. The mechanistic shift was accompanied by parallel changes in gender distribution, with an observable increase in female predominance after the transition point.

Therapeutic efficacy by dominant mechanism showed significant differences in treatment response. Acetazolamide demonstrated largest effect in venous-dominant IIH (Hedges' g = 1.86, NNT = 2.4) but much lower efficacy in obesity-dominant IIH (g = 0.72, NNT = 6.8). However, weight management interventions showed greatest benefit in obesity-dominant IIH (g = 1.75, NNT = 2.7) but limited efficacy in venous-dominant presentations (g = 0.62, NNT = 7.2). Vitamin D supplementation was most effective in hormone-dominant cases (g = 1.64, NNT = 2.8).

Based on these findings, an evidence-based precision treatment algorithm was developed, stratifying by age and dominant pathophysiological mechanism. This mechanism-targeted approach demonstrated 63% improved outcomes (95% CI: 47-79%, P-value<0.001) compared to standard care across all IIH phenotypes, with greater benefits observed in clearly defined mechanistic subgroups.

## 4 Discussion

Pediatric IIH has been viewed as a uniform condition across childhood, with management strategies largely extrapolated from adult studies. However, mounting evidence suggests that the pathophysiological mechanisms underlying IIH in children may vary significantly with age and developmental stage. The parallel epidemiological trends observed between pediatric IIH and growth plate disorders, especially their shared associations with obesity and hormonal factors, suggest possible common underlying mechanisms that have remained largely unexplored (Esmaili and Bradfield, 2007; Riedel et al., 2024; Alswaina et al., 2015).

Understanding these age-dependent pathophysiological differences is important for optimizing management strategies in pediatric populations. While adult IIH demonstrates clear associations with obesity, especially in women of childbearing age, pediatric cases show more complex patterns with different risk factors and presentations across various age groups (Azzam et al., 2024c, 2024d).

Through SEM, we demonstrated that prepubertal children show leading venous pressure and hormonal pathway contributions to IIH development, while postpubertal patients demonstrated obesity-dominant mechanisms. Our results revealed that mechanism-specific management achieved 63% better outcomes compared to standard care, with acetazolamide and surgical interventions proving most effective for venous-dominant cases, weight management strategies showing superior results in obesity-dominant phenotypes, and vitamin D supplementation demonstrating significant benefit specifically in hormonal-dominant presentations.

We also identified four peculiar clinical phenotypes with age-dependent distributions: Classical (mostly postpubertal), Atypical (more common prepubertally), Vision-threatened, and Cranial nerve presentations. Our findings demonstrated significant differences in population attributable risk for obesity between age groups (35.7% prepubertal versus 78.6% postpubertal) and formulated clear correlations between growth plate disorders and shared risk factors including obesity and hormonal dysregulation.

The identification of prepubertal dominance by venous pressure and hormonal pathways, contrasted with postpubertal obesity-driven mechanisms, provides us with a framework for understanding why certain children develop IIH and others do not. This mechanistic understanding explains the puzzling observation that obesity shows weaker associations with IIH in younger children compared to adolescents (Lyons et al., 2023).

From a clinical perspective, our discovery that prepubertal children are more likely to present asymptomatically estimated at 12.8% versus 3.2% in postpubertal patients, and with cranial nerve palsies suggests that screening strategies should differ by age group. The higher prevalence of diplopia in younger children, estimated at 47.9% versus 32.6%, versus blurred vision in older children for 56.9% versus 22.4%, provides practical guidance for symptom assessment and parent education.

We found that CSF opening pressure was significantly higher in postpubertal children with 372 cmH_2_O versus 305 cmH_2_O, with stronger correlations between BMI and CSF pressure in older children. This finding has important highlights and considerations that we should expect lower opening pressures in younger children and should not dismiss the diagnosis based on the baseline normal adult-derived pressure thresholds. Our identification of lower CSF protein levels in prepubertal patients with 0.201 g/L versus 0.253 g/L, suggests increased CSF production rather than impaired drainage as the primary mechanism in younger children.

The elevated venous pressures we documented in prepubertal patients as in superior sagittal sinus pressure 18.9 mmHg, normal 2-10 mmHg, provide objective evidence for venous-mediated mechanisms. This finding supports early consideration of venographic evaluation in younger children, especially when other risk factors are absent.

Our analysis revealed that obesity contributes to only 35.7% of IIH cases in children under ten years old, compared to 78.6% in older children. This significant difference brings to our attention that weight management should be the primary focus in postpubertal patients, while alternative mechanisms should be investigated in younger children. The threshold effects we identified as BMI z-score 1.64 for IIH, 1.22 for growth plate disorders, provide possible cutoff points to be considered for risk assessment.

The superior efficacy of weight management interventions in obesity-dominant phenotypes estimated at 75% improvement versus 27% standard care, validates this stratified approach. For postpubertal patients, our findings support aggressive weight management as first-line therapy, while prepubertal patients require more stratified and structured evaluation of alternative mechanisms.

Our identification of shared risk factors between IIH and growth plate disorders, including biomechanical stress observations and hormonal impacts, provides novel highlights into the systemic effects of the underlying pathophysiology. The higher estimated shear stress in obese children evident by 1.47 MPa versus 0.82 MPa in normal weight, demonstrates how obesity creates mechanical vulnerability beyond its metabolic effects.

The geographical and seasonal variations we observed for both conditions support vitamin D-mediated mechanisms. Our finding of higher incidence rates in northern latitudes provides a recommendation for vitamin D screening, especially in prepubertal patients where hormonal pathways are mostly concerning (Nikooyeh et al., 2021; Roth et al., 2018; Heidari and Haji Mirghassemi, 2012).

We demonstrated that vitamin D deficiency affects over one-quarter of pediatric IIH patients, with higher prevalence in prepubertal children at 35.3% versus 18.9%. The specific efficacy of vitamin D supplementation in hormonal-dominant cases with 64% improvement validates targeted intervention approaches. Our findings suggest that all prepubertal IIH patients should undergo hormonal evaluation, including vitamin D, growth hormone status, and seasonal factors.

The significantly elevated IIH risk with growth hormone therapy that is significantly evident by 30-fold increase requires careful consideration. Our findings suggest that children receiving growth hormone therapy need closer monitoring, especially those with additional risk factors like renal insufficiency where we found 147-fold increased risk.

We identified four phenotypes with age-dependent distributions: Classical IIH that is more common postpubertally, Atypical presentations that is prepubertally dominant, Vision-threatened, and Cranial nerve variants. This phenotypic classification provides us with a framework for anticipating presentations and guiding diagnostic workup based on age and symptom observation.

The higher prevalence of severe papilledema in postpubertal patients estimated at 54.6% versus 18.5%, suggests that younger children may require different ophthalmologic monitoring protocols. The more frequent occurrence of empty sella as 60.5% versus 25.6%, and transverse sinus stenosis as 82.6% versus 41.3%, in older patients provides imaging guidance for radiologists.

Our demonstration of 63% improved outcomes with mechanism-specific therapy compared to standard care provides quantitative validation for precision medicine approaches in pediatric IIH. The peculiar efficacy patterns we identified, acetazolamide best for venous-dominant cases, weight management optimal for obesity-dominant presentations, vitamin D supplementation effective specifically for hormonal-dominant disease, provide structured evidence-based guidelines that shall be further trialed and studied.

The number needed to treat calculations we derived with 2.4 for acetazolamide in venous-dominant cases, 2.7 for weight management in obesity-dominant cases, 2.8 for vitamin D in hormonal-dominant cases, provide us with quantitative expectations for therapeutic response.

Our study faces several important limitations that must be acknowledged when interpreting these findings. First, we were constrained by the limited number of high-quality pediatric IIH studies available in the literature. With only 22 studies meeting our inclusion criteria from an initial search of 1571 records, the pediatric IIH studies remain relatively lower compared to adult studies. This scarcity reflects the lower incidence of pediatric IIH compared to adult cases, making large-scale studies challenging to conduct (Zhou et al., 2024; Alqahtani et al., 2023).

The heterogeneity in diagnostic criteria across studies presented another significant limitation. While we required studies to use established diagnostic criteria as, modified Dandy criteria, Friedman criteria, or other standards, the progression of these criteria over time and variations in implementation across different centers introduced a risk of variability in IIH definitions. This heterogeneity may have affected our ability to detect some of mechanistic differences or contributed to the residual heterogeneity we observed in some analyses.

Our use of SEM, while innovative for this application, comes with some limitations that we must acknowledge. SEM requires large sample sizes to achieve adequate statistical power, and while our total sample of 2293 participants was significant, the subdivision into age-stratified groups reduced power for detecting smaller effect sizes. The assumption of multivariate normal distribution required by SEM may not hold for all variables in our model, especially for categorical outcomes and skewed continuous variables like CSF opening pressure.

We conducted primarily cross-sectional analyses due to the nature of available data, which limits our ability to establish temporal relationships between variables. The developmental transition we identified occurs over months to years, but our analysis treats it as a discrete cutoff point. This limitation is important when considering the pubertal transition, which varies significantly between individuals and may not align precisely with chronological age.

Our included studies showed geographic clustering, with the majority originating from North America and Europe. This geographic limitation may affect the generalizability of our findings to different populations from all over the world, especially those with different genetic backgrounds, environmental exposures, or healthcare systems. The relationship between vitamin D deficiency and IIH, for example, may vary significantly across populations with different sun exposure observations, dietary habits, and genetic variants affecting vitamin D metabolism.

We also acknowledge the risk of selection bias in our included studies, as most came from tertiary care centers that may see more severe or complex cases. This referral bias could affect our estimates of disease phenotype prevalence and treatment efficacy. Community-based studies of pediatric IIH are extremely rare, limiting our understanding of the full spectrum of disease presentation and outcomes.

Despite our dedicated literature search strategy and inclusion of multiple databases, we detected evidence of publication bias for certain pathways in our study. Small studies with negative results are less likely to be published, especially in pediatric populations where recruitment is challenging. Our trim-and-fill adjustments and PET-PEESE corrections addressed this bias to some extent, but residual effects likely remain.

The quality of included studies varied, with risk of bias assessment quality scores ranging from five to nine. While we conducted sensitivity analyses excluding lower-quality studies, this approach reduced our already limited sample size. The retrospective nature of most included studies also introduces risk of information bias, especially for variables like BMI history or symptom onset timing.

Our analysis of growth plate disorders was limited by the relative scarcity of studies simultaneously reporting both IIH and growth plate disorder data. Most of our growth plate disorder findings were derived from separate studies investigating these conditions independently, limiting our ability to directly assess shared mechanisms in the same patient populations. The indirect nature of these relationships, while statistically significant, requires validation in studies specifically designed to look for both conditions simultaneously.

Our findings highlight the need for longitudinal studies that follow children through the pubertal transition to capture the dynamic nature of pathophysiological mechanism changes. We recommend initiating multicenter prospective cohorts that begin enrollment in prepubertal children and follow participants through adolescence, with regular assessments of hormonal status, growth patterns, body composition, and intracranial pressure markers. Such studies should include detailed pubertal staging using Tanner criteria rather than relying only on chronological age.

These longitudinal studies shall integrate advanced neuroimaging techniques, including quantitative MRI measures of venous sinus caliber, CSF flow dynamics, and brain volume changes. Serial lumbar punctures may not be feasible for research purposes, but non-invasive intracranial pressure monitoring techniques under development could provide valuable longitudinal data (Burman et al., 2019).

Based on our findings of the proposed pathophysiological mechanisms, we recommend focused studies on developing practical biomarker panels that can identify mechanism-dominant phenotypes in practice. Priority biomarkers should include comprehensive hormonal panels including, vitamin D, growth hormone, IGF-1, sex hormones, inflammatory markers, and metabolic factors that can be easily obtained in routine care (Triana et al., 2024; Müller et al., 2023; Pham and Ng, 2024).

We should focus on developing and validating simplified risk scoring systems that combine readily available clinical data as age, sex, BMI, and symptoms, with targeted biomarker testing to predict mechanism dominance and guide treatment selection. Machine learning solutions may be valuable for identifying complex interaction patterns between clinical and laboratory variables.

Our demonstration of mechanism-specific therapeutic efficacy provides a foundation for designing targeted intervention trials. We recommend further clinical trials comparing mechanism-targeted therapy versus standard care, with stratification by predicted mechanism dominance. For prepubertal children, studies should evaluate hormonal optimization including vitamin D repletion, growth hormone monitoring protocols, and evaluation of anti-inflammatory approaches.

For postpubertal patients, studies should focus on better weight management strategies, including evaluation of newer pharmacological approaches like GLP-1 receptor agonists specifically in pediatric IIH populations. Investigation of combination management strategies targeting multiple pathways simultaneously may result in superior outcomes, especially during transitional periods where multiple mechanisms may be operative.

The rarity of pediatric IIH necessitates international collaborative efforts to achieve adequate sample sizes for definitive studies. We recommend developing international pediatric IIH research consortiums that standardize diagnostic criteria, data collection protocols, and outcome measures across participating centers. Such networks could facilitate rapid patient accrual for intervention studies and enable investigation of rare phenotypes or treatment-resistant cases.

These collaborative networks should prioritize diversity in geographic representation, socioeconomic status, and ethnic backgrounds to ensure findings are generalizable across populations. Special attention should be paid to recruiting from underrepresented populations that may have different risk profiles or treatment responses.

Our findings require translation into clinical practice through implementation science evidence. Studies should evaluate the feasibility, acceptability, and effectiveness of mechanism-based diagnostic protocols in practice settings. Barriers to implementation may include personnel training needs, resource requirements for comprehensive assessment, and healthcare system adaptations required to support precision medicine approaches.

Cost-effectiveness analyses should evaluate whether the improved outcomes achieved with mechanism-targeted therapy justify the additional diagnostic evaluation required. These analyses should consider both short-term treatment costs and long-term outcomes including visual preservation, quality of life, and prevention of treatment complications.

Future studies shall optimally include advanced recent technologies to improve pediatric IIH diagnosis and management. Non-invasive intracranial pressure monitoring techniques, advanced neuroimaging protocols for mechanism identification, and machine learning-based approaches for pattern recognition all show promise for promoting for better precision medicine implementation.

## 5 Conclusion

Our systematic review and meta-analysis views pediatric IIH as a developmentally heterogeneous condition with age-dependent pathophysiological mechanisms rather than a uniform disease entity. We have found that prepubertal children mostly has venous pressure and hormonal pathway-driven disease, while postpubertal patients demonstrate obesity-dominant mechanisms, with precise developmental crossover points at 10.2 years for females and 11.4 years for males. Most importantly, we demonstrated that mechanism-specific therapeutic approaches achieve 63% superior outcomes compared to standard care, providing quantitative evidence that precision medicine strategies can improve clinical outcomes in pediatric IIH management.

These findings necessitate a moving step from age-agnostic treatment protocols toward mechanism-based therapeutic selection that considers developmental stage, hormonal status, and individual risk factor profiles. Our work formulates the foundation for evidence-based precision medicine for next studies about pediatric IIH, with immediate clinical applications including hormonal evaluation for prepubertal patients, aggressive weight management for postpubertal cases, and targeted interventions based on mechanistic phenotyping. The significant improvement in outcomes achieved through our proposed framework resonates the transition toward personalized care strategies and highlights the need for implementation evidence, international collaborative networks, and longitudinal studies to fully realize the role of mechanism-targeted therapy in preventing the devastating visual and neurological complications of the disease.

## Institutional review board (IRB) approval

Not applicable. This study was a systematic review and meta-analysis of previously published data and did not require IRB approval.

## Consent for publication

Not applicable.

## CRediT authorship contribution statement

**Yazan Jumah Alalwani:** Conceptualization, Data curation, Formal analysis, Investigation, Methodology, Writing – original draft. **Eyad S. Alhashim:** Data curation, Formal analysis, Investigation, Writing – original draft. **Layan Nahar Alqahtani:** Data curation, Investigation, Validation, Writing – review & editing. **Reem Mohammed Albogami:** Data curation, Investigation, Validation, Writing – review & editing. **Khawlah Abdullah Ali Almana:** Data curation, Investigation, Writing – review & editing. **Abdullah Saeed Raffaa:** Data curation, Investigation, Writing – review & editing. **Ewan Saad Alomar:** Data curation, Investigation, Writing – review & editing. **Ammar Mohammed Alnujaidi:** Data curation, Investigation, Writing – review & editing. **Omar Gharamallah Alghamdi:** Data curation, Investigation, Writing – review & editing. **Abdulaziz Saad Alomar:** Data curation, Investigation, Writing – review & editing. **Abdullah Zahim A. Alotaibi:** Data curation, Investigation, Writing – review & editing. **Zainab Saeed Alwusaybie:** Data curation, Investigation, Writing – review & editing. **Mustafa S. Alhasan:** Formal analysis, Software, Visualization, Writing – review & editing. **Ahmed Y. Azzam:** Conceptualization, Formal analysis, Methodology, Project administration, Resources, Software, Supervision, Visualization, Writing – original draft, Writing – review & editing.

## Financial disclosures

None of the authors have any financial relationships or disclosures relevant to this study.

## Declaration of competing interest

The authors declare that they have no known competing financial interests or personal relationships that could have appeared to influence the work reported in this paper.

## Data Availability

No new data were generated for this systematic review. All data analyzed were extracted from previously published studies cited in the references.
