# Supplementary material for: Developmental transition in pathophysiological mechanisms of pediatric idiopathic intracranial hypertension and growth plate disorders: A multi-level structural equational modeling systematic review and meta-analysis
Source: Brain Spine. 2026 Jul 10;6:106164. doi: 10.1016/j.bas.2026.106164 (PMC13427536; doi:10.1016/j.bas.2026.106164)
Supplement: Multimedia component 1 [file mmc1.docx]

**Supplementary Table 1:** Publication Bias Assessment, Sensitivity Analysis, and Statistical Corrections.

| **Method** | **Obesity→IIH Pathway** | **Venous→IIH Pathway** | **Hormonal→IIH Pathway** | **Obesity→GPD Pathway** | **Statistical Significance** | **Methodological Notes** |
| --- | --- | --- | --- | --- | --- | --- |
| **PUBLICATION BIAS ASSESSMENT** | | | | | | |
| Egger's regression test | Intercept = 0.86  (95% CI: −0.17-1.89)  P-value= 0.079 | Intercept = 1.24  (95% CI: 0.29-2.19)  P-value= 0.014 | Intercept = 0.52  (95% CI: −0.46-1.50)  P-value= 0.276 | Intercept = 1.92  (95% CI: 0.84-3.00)  P-value= 0.003 | Significant bias detected for venous and obesity→GPD pathways | Standard precision-based method; sensitivity 0.72, specificity 0.84 in simulation studies |
| Begg-Mazumdar test | τ = 0.21  P-value = 0.087 | τ = 0.33  P-value= 0.012 | τ = 0.18  P-value= 0.142 | τ = 0.39  P-value= 0.008 | Consistent with Egger's test | Rank correlation method based on Kendall's tau; less power than Egger's but more robust to outliers |
| Trim-and-fill method | Adjusted β = 0.69  (95% CI: 0.56-0.82)  Δ = −0.07 (−9.2%) | Adjusted β = 0.53  (95% CI: 0.41-0.65)  Δ = −0.15 (−22.1%) | Adjusted β = 0.31  (95% CI: 0.21-0.41)  Δ = −0.06 (−16.2%) | Adjusted β = 0.61  (95% CI: 0.49-0.73)  Δ = −0.17 (−21.8%) | Modest adjustments for obesity→IIH  Significant for venous and obesity→GPD | Imputes possibly missing studies to create a symmetric distribution; non-parametric approach |
| PET-PEESE correction | Adjusted β = 0.72  (95% CI: 0.61-0.83)  Δ = −0.04 (−5.3%) | Adjusted β = 0.56  (95% CI: 0.43-0.69)  Δ = −0.12 (−17.6%) | Adjusted β = 0.33  95% CI: 0.23-0.43)  Δ = −0.04 (−10.8%) | Adjusted β = 0.63  (95% CI: 0.51-0.75)  Δ = −0.15 (−19.2%) | Consistent with trim-and-fill | Precision-Effect Test and Precision-Effect Estimate with Standard Error; meta-regression approach |
| p-curve analysis | Right-skewed  Z = 8.91, P-value< 0.001  Power = 94% | Right-skewed  Z = 6.37, P-value < 0.001  Power = 88% | Right-skewed  Z = 5.72, P-value < 0.001  Power = 79% | Right-skewed  Z = 7.14, P-value< 0.001  Power = 92% | No evidence of p-hacking or selective reporting | Distribution analysis of significant p-values; tests evidential value and selective reporting |
| **SENSITIVITY ANALYSES** | | | | | | |
| Leave-one-out analysis | Range: 0.68-0.78  CV = 4.3%  Min P-value < 0.001 | Range: 0.54-0.73  CV = 8.7%  Min P-value< 0.001 | Range: 0.27-0.39  CV = 12.1%  Min P-value = 0.002 | Range: 0.65-0.83  CV = 7.2%  Min P-value< 0.001 | All pathways remain significant under all iterations | Systematic removal of each study; CV = coefficient of variation; stability metric |
| Study quality stratification | High quality (n= 12):  β = 0.75 (0.65-0.85)  Low quality (n= 10):  β = 0.72 (0.59-0.85)  Δ = 0.03, P-value= 0.694 | High quality (n= 12):  β = 0.70 (0.59-0.81)  Low quality (n=10):  β = 0.64 (0.49-0.79)  Δ = 0.06, P-value= 0.512 | High quality (n=12):  β = 0.34 (0.23-0.45)  Low quality (n= 10):  β = 0.33 (0.20-0.46)  Δ = 0.01, P-value= 0.901 | High quality (n= 12):  β = 0.81 (0.71-0.91)  Low quality (n= 10):  β = 0.74 (0.61-0.87)  Δ = 0.07, P-value= 0.402 | No significant effect of study quality on any pathway | Quality assessed using modified Newcastle-Ottawa Scale; cutoff at median score (7) |
| Sample size stratification | Large (n= 11):  β = 0.73 (0.63-0.83)  Small (n= 11):  β = 0.77 (0.64-0.90)  Δ = −0.04, P-value= 0.623 | Large (n= 11):  β = 0.66 (0.56-0.76)  Small (n= 11):  β = 0.72 (0.58-0.86)  Δ = −0.06, P-value= 0.456 | Large (n=11):  β = 0.30 (0.21-0.39)  Small (n=11):  β = 0.38 (0.26-0.50)  Δ = −0.08, P-value= 0.308 | Large (n= 11):  β = 0.77 (0.67-0.87)  Small (n= 11):  β = 0.81 (0.68-0.94)  Δ = −0.04, P-value= 0.618 | No significant small-study effects detected | Cutoff at median sample size (n=57); small studies do not systematically overestimate effects |
| Diagnostic criteria (IIH) | Friedman criteria (n= 8):  β = 0.76 (0.64-0.88)  Modified Dandy (n= 9):  β = 0.71 (0.60-0.82)  Δ = 0.05, P-value= 0.538 | Friedman criteria (n= 8):  β = 0.68 (0.57-0.79)  Modified Dandy (n= 9):  β = 0.66 (0.54-0.78)  Δ = 0.02, P-value = 0.804 | Friedman criteria (n= 8):  β = 0.35 (0.24-0.46)  Modified Dandy (n= 9):  β = 0.32 (0.21-0.43)  Δ = 0.03, P-value = 0.699 | N/A | No significant effect of diagnostic criteria | Consistent findings across diagnostic criteria generations |
| GPD classification (SCFE) | Early/pre-slip (n= 7):  β = 0.79 (0.66-0.92)  Established SCFE (n= 7):  β = 0.74 (0.61-0.87)  Δ = 0.05, P-value= 0.602 | N/A | N/A | N/A | No significant effect of SCFE classification | Findings significant to severity classification systems |
| **STATISTICAL CORRECTIONS** | | | | | | |
| Multiple testing adjustment | Original P-value < 0.001  Bonferroni P-value< 0.001  FDR q < 0.001 | Original P-value< 0.001  Bonferroni P-value < 0.001  FDR q < 0.001 | Original P-value = 0.002  Bonferroni P-value = 0.026  FDR q = 0.008 | Original P-value < 0.001  Bonferroni P-value < 0.001  FDR q < 0.001 | All pathways remain significant after correction | Bonferroni (family-wise error control) and Benjamini-Hochberg false discovery rate correction applied |
| Heterogeneity adjustment | Original I² = 36.4%  β = 0.76 (0.67-0.85)  Adjusted β = 0.75 (0.63-0.87) | Original I² = 42.1%  β = 0.68 (0.54-0.82)  Adjusted β = 0.67 (0.49-0.85) | Original I² = 28.7%  β = 0.37 (0.27-0.47)  Adjusted β = 0.36 (0.25-0.47) | Original I² = 39.5%  β = 0.78 (0.68-0.88)  Adjusted β = 0.77 (0.64-0.90) | Minimal impact on effect estimates | Hartung-Knapp-Sidik-Jonkman method; robust variance estimation for random-effects models |
| Measurement error correction | Original β = 0.76  Disattenuated β = 0.83  Δ = +0.07 (+9.2%) | Original β = 0.68  Disattenuated β = 0.77  Δ = +0.09 (+13.2%) | Original β = 0.37  Disattenuated β = 0.43  Δ = +0.06 (+16.2%) | Original β = 0.78  Disattenuated β = 0.86  Δ = +0.08 (+10.3%) | Stronger effects after correction | Correction using reliability coefficients (ω); addresses measurement attenuation |
| Small-sample bias correction | Original β = 0.76  Corrected β = 0.74  Δ = −0.02 (−2.6%) | Original β = 0.68  Corrected β = 0.65  Δ = −0.03 (−4.4%) | Original β = 0.37  Corrected β = 0.35  Δ = −0.02 (−5.4%) | Original β = 0.78  Corrected β = 0.76  Δ = −0.02 (−2.6%) | Minimal impact on effect estimates | Switzer small-sample bias correction; addresses upward bias in small samples |
| **SIGNIFICANCE SUMMARY** | | | | | | |
| Least conservative estimate | β = 0.83 (0.73-0.93) | β = 0.77 (0.67-0.87) | β = 0.43 (0.32-0.54) | β = 0.86 (0.76-0.96) | All pathways remain significant | Disattenuated for measurement error |
| Most conservative estimate | β = 0.69 (0.56-0.82) | β = 0.53 (0.41-0.65) | β = 0.30 (0.19-0.41) | β = 0.61 (0.49-0.73) | All pathways remain significant | Trim-and-fill adjustment for publication bias |
| Average adjusted estimate | β = 0.74 (0.65-0.83) | β = 0.63 (0.51-0.75) | β = 0.34 (0.24-0.44) | β = 0.72 (0.62-0.82) | All pathways remain significant | Meta-analytic average of all correction methods |
| Failure threshold | Nullification: n= 68  Significance: n= 12 | Nullification: n= 54  Significance: n= 9 | Nullification: n= 26  Significance: n= 4 | Nullification: n= 61  Significance: n= 11 | High significance to missing studies | Rosenthal's fail-safe N; number of studies needed to nullify effect or significance |

**Abbreviations**: SEM = Structural equation modeling; IIH = Idiopathic intracranial hypertension; GPD = Growth plate disorders; SCFE = Slipped capital femoral epiphysis; β = Standardized path coefficient; CI = Confidence interval; Δ = Change in estimate; CV = Coefficient of variation; FDR = False discovery rate; I² = Measure of heterogeneity; p = Probability value; τ = Kendall's tau; N/A = Not applicable.
